# Dysregulation of SRSF3/*circSAMD4*/CIRBP Axis Promotes Iodinated Contrast-induced Acute Kidney Injury

**DOI:** 10.7150/ijbs.117838

**Published:** 2025-09-29

**Authors:** Xi Wu, Ting Wu, Xiufen Wang, Meiyu Zeng, Chengyuan Tang, Juan Cai, Anqun Chen, Guochun Chen, Zhiwen Liu, Yu Liu, Shaobin Duan

**Affiliations:** Department of Nephrology, The Second Xiangya Hospital of Central South University; Hunan Key Laboratory of Kidney Disease and Blood Purification, Changsha 410011, China.

**Keywords:** Iodinated contrast media, acute kidney injury, *circSAMD4*, CIRBP, SRSF3

## Abstract

Iodinated contrast agents are a common cause of contrast-induced acute kidney injury (CI-AKI), yet the underlying mechanisms remain unclear. We found that* circSAMD4* is markedly upregulated in renal tubular epithelial cells (RTECs) from iohexol-induced CI-AKI mice and patients diagnosed with acute tubular injury (ATI). Silencing *circSAMD4* alleviated kidney injury and tubular cell death in CI-AKI mice, whereas its overexpression promoted apoptosis in iohexol-treated RTECs. Mechanistically, *circSAMD4* binds to cold-inducible RNA-binding protein (CIRBP) and inhibits its nuclear import. Renal tubule-specific *Cirbp* deletion mitigated CI-AKI, while CIRBP overexpression abolished the protective effects of *circSAMD4* knockdown against iohexol-induced apoptosis. *CircSAMD4* upregulation in iohexol-treated RTECs was driven by serine/arginine-rich splicing factor 3 (SRSF3) downregulation. Similar molecular alterations in clinical samples correlated with kidney function decline. These findings identify the SRSF3/*circSAMD4*/CIRBP axis as a novel pathogenic mechanism in CI-AKI and highlight *circSAMD4* as a promising therapeutic target.

## Introduction

Iodinated contrast-induced acute kidney injury (CI-AKI) is defined as a rapid decline in renal function following the intravascular administration of iodinated contrast media, after excluding other potential causes 1. This kind of disease represents a significant healthcare burden, accounting for approximately 11% of hospitalized AKI cases 2. Large-scale randomized controlled trials have demonstrated that traditional therapies, including sodium bicarbonate and oral N-acetylcysteine have not shown significant benefits in patients with CI-AKI 3. Previous studies have suggested that iodinated contrast media contribute to renal injury by altering renal hemodynamics and directly inducing damage or apoptosis in renal tubular epithelial cells (RTECs) 4. However, the precise pathogenic mechanisms underlying CI-AKI remain poorly understood. Thus, elucidating these molecular mechanisms is greatly beneficial for identifying novel therapeutic targets.

Circular RNAs (circRNAs), a class of noncoding RNAs, are characterized by a single-stranded, covalently closed-loop structure with high stability and conservation in mammalian cells 5, 6. This kind of RNA has diverse biological functions, including acting as microRNA and protein sponges 7-9, serving as scaffolds for enzyme-substrate interactions 10, and functioning as templates for protein translation 11-13. Emerging evidence indicates that circRNAs play critical regulatory roles in kidney diseases 14-16. In AKI models induced by sepsis or ischemia-reperfusion injury (IRI), aberrant circRNA expression has been implicated in pathological cascades such as dysregulated intracellular signaling, oxidative stress, apoptosis, excessive inflammation, and tissue damage 16. Although circRNA dysregulation has also been reported in drug-induced AKI, its pathogenic mechanisms remain largely unexplored 16. In our previous study using an iohexol-induced CI-AKI rat model, we identified 38 differentially expressed circRNAs compared to saline-treated controls, highlighting altered circRNA-associated competing endogenous RNA (ceRNA) networks 17. Subsequently, another study reported that circRNA-associated ceRNA networks were also involved in the protective effects of icariin in CI-AKI 18. However, the critical circRNAs that participate in CI-AKI pathogenesis remain elusive.

Cold-inducible RNA-binding protein (CIRBP) is a glycine-rich RNA-binding protein initially identified for its role in cold stress 19. CIRBP regulates various cellular processes, including proliferation and apoptosis 20. Under stress conditions, such as mild cold shock, ultraviolet irradiation, or hypoxia, CIRBP relocates from the nucleus to the cytoplasm, stabilizing specific mRNAs and promoting their translation 21. Emerging evidence implicates CIRBP in renal pathophysiology and AKI. In IRI-induced AKI, CIRBP exacerbates tissue damage by modulating inflammatory and oxidative responses 22, whereas a CIRBP-derived peptide can attenuate inflammation in septic AKI 23. Conversely, CIRBP may protect the kidney after hypothermic cardiovascular surgery by suppressing PHD3/HIF-1α signaling 24. However, its role in CI-AKI remains unexplored.

Serine/arginine-rich splicing factor 3 (SRSF3) is an evolutionarily conserved RNA-binding protein that primarily regulates alternative splicing 25. In Drosophila, SR proteins have been shown to inhibit circular RNA production, such as Laccase2 circRNA 26. Recent studies highlight SRSF3 as a key regulator of both N6-methyladenosine (m6A) modifications in long non-coding RNAs and circRNAs 27, 28. Functionally, SRSF3 has been implicated in liver diseases, ischemic stroke, and acute myocardial infarction 29-31. However, its role in AKI has not yet been explored.

In this study, we demonstrated that upregulation of *circSAMD4* exacerbated iodinated contrast-induced cell apoptosis through inhibiting nuclear import of CIRBP, while downregulation of SRSF3 mediated the increase of *circSAMD4* expression in RTECs. Notably, these molecular alterations were also observed in RTECs from patients with biopsy-confirmed acute tubular injury (ATI), where their dysregulated expression correlated with kidney dysfunction. Collectively, these findings establish the dysregulation of the SRSF3/*circSAMD4*/CIRBP axis as a novel pathogenic mechanism in CI-AKI and highlight *circSAMD4* as a potential therapeutic target.

## Methods

### Establishment of CI-AKI mice model and and cisplatin-induced AKI mice model

**Figure 1:**
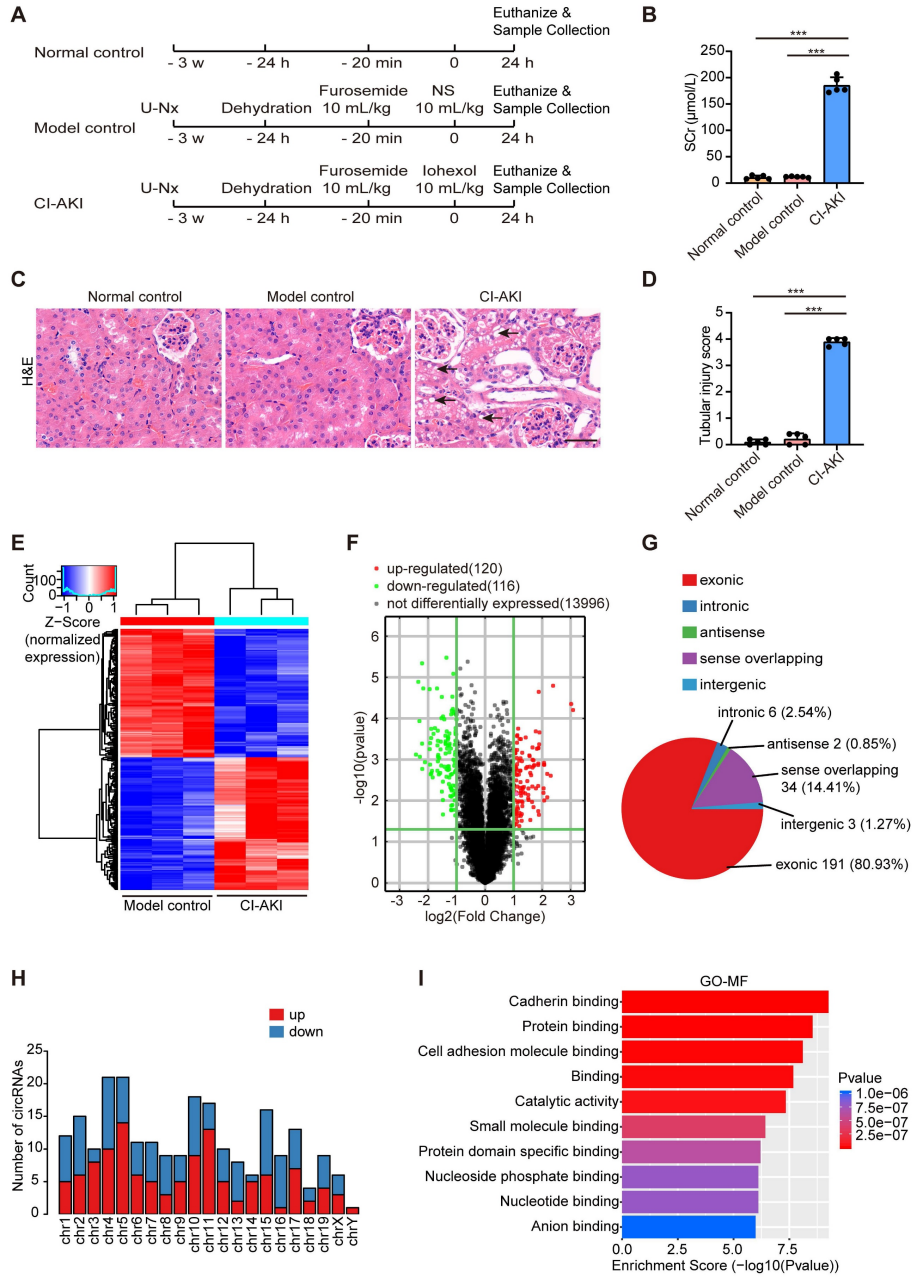
** Differential circRNA expression profiles in a CI-AKI mouse model.** Male C57BL/6J mice were subjected to the following procedure: unilateral nephrectomy (U-Nx) with a 3-week recovery period, followed by sequential interventions of 24-hour water deprivation, tail vein injection of furosemide (10 mL/kg), and after 20 minutes, tail vein administration of either iohexol (10 mL/kg; CI-AKI group) or normal saline (NS; model control group). Serum and kidney tissues were harvested 24 hours after the final injection. (A) Schematic illustration of experimental groups: CI-AKI, model control, and normal control. (B) Serum creatinine (SCr) levels (n = 5). (C and D) Representative images of hematoxylin and eosin (H&E) staining and pathological score in kidney tissues. The black arrow indicated vacuolar degeneration of tubular epithelial cells (n = 5). Scale bar: 50 μm. (E and F) Hierarchical clustering and volcano plot analysis of differentially expressed circRNAs in kidney samples from model control and CI-AKI groups using Arraystar Mouse CircRNA Microarray (cutoff criteria: |log2 fold change| > 1, *P* < 0.05). (G) Distribution of genomic origins of the 236 differentially expressed circRNAs. (H) Chromosomal distribution of differentially expressed circRNAs. (I) Gene Ontology (GO) analysis showing enriched molecular functions (MF) for the host genes of upregulated circRNAs. All quantitative data are presented as mean ± SD (B and D). Statistical analysis was performed using one-way ANOVA with Tukey's multiple comparisons test (B and D). ***P < 0.001.

The Animal Ethical and Welfare Committee of Central South University approved the protocols used in all animal investigation (approval number: CSU-2023-0021). C57BL/6 mice (6-8 weeks, male) were bred in the Experimental Animal Center of Central South University and were fed under specific pathogen-free conditions. The *in vivo* induction method for CI-AKI, adapted from previous studies 32-35, is illustrated in Figure 1A. Briefly, following administration of pentobarbital (60 mg/kg), mice received right nephrectomy. Three weeks post-surgery, the following interventions were performed sequentially: 24 h water deprivation, intravenous administration of furosemide (10 mL/kg; Harvest Pharmaceutical, Shanghai, China), followed by iohexol (10 mL/kg; Omnipaque, 350 mg iodine/mL; GE Healthcare, Shanghai, China) or normal saline via tail vein injection after a 20-min interval. Mice were euthanized 24 h after iohexol or saline administration for the collection and storage of serum and kidney tissues.

For cisplatin-induced AKI mice model, C57BL/6 mice (8 weeks, male) were injected by i.p. injection with a single dose of cisplatin at 30 mg/kg 36. Control mice were injected with normal saline. Serum and kidney tissues were collected at 48 hours after cisplatin injection.

### Analysis of renal function and histopathology

Renal function was evaluated by detecting serum creatinine (SCr) levels using an automatic biochemical analyzer at the Department of Clinical Laboratory Medicine. Histological examination was performed to assess tubular injury. Kidney tissues were processed by fixation in 4% paraformaldehyde, followed by paraffin embedding and sectioning at a thickness of 4 μm for hematoxylin and eosin (H&E) staining. Tubular injury was evaluated by examining at least ten randomly selected fields from the renal cortex and outer medulla. The extent of tubular injury was graded on a scale of 0 to 4, according to the percentage of damaged tubules: 0 (no damage), 1 (<25%), 2 (25-50%), 3 (50-75%), and 4 (>75%) 37.

### Microarray analysis

Total RNAs were extracted from kidney cortex of mice with or without iohexol treatment. The samples were prepared and microarray hybridization was processed in line with Arraystar's protocols (KangChen Biotech, Shanghai, China). Briefly, total RNAs were subjected to RNase R exonuclease, leading to the elimination of linear RNAs and enrichment of circRNAs. Subsequently, the enriched circRNAs were amplified and transcribed into fluorescent cRNA using a random priming method (Arraystar Super RNA Labeling Kit; Arraystar). The labeled cRNAs were hybridized onto the Arraystar Mouse circRNA Array v2 (8x15K, Arraystar). The array images were scanned using an Agilent Scanner G2505C and analyzed with Agilent Feature Extraction software (version 11.0.1.1). R software limma package was employed for subsequent data processing. The identification of dysregulated expressed circRNAs was conducted using dual criteria: |log2 fold change| > 1 and *P* < 0.05.

### RNA sequencing

Total RNAs were extracted from iohexol-induced proximal RTEC line (HK-2 cells) after treatments and underwent quality assessment. For sequencing library preparation, mRNA was purified from total RNA using poly-T oligo-attached magnetic beads. Fragmentation was carried out using divalent cations in an Illumina proprietary buffer. First-strand cDNA was synthesized using random hexamers and SuperScript II, followed by second-strand synthesis with DNA Polymerase I and RNase H. Remaining overhangs were converted to blunt ends using exonuclease/polymerase activities. After end repair and A-tailing, Illumina PE adapter oligonucleotides were ligated to the cDNA fragments. The library was purified using the AMPure XP system (Beckman Coulter) and enriched by PCR. The final library was quantified using the Agilent high sensitivity DNA assay on a Bioanalyzer 2100 (Agilent). Sequencing was performed on the NovaSeq 6000 platform (Illumina) by Personalbio (Shanghai, China).

### Gene Ontology (GO) analysis

The GO project provides a controlled vocabulary to describe gene and gene product attributes in any organism (http://www.geneontology.org) 38. GO analysis was performed using the R package TopGO (version 2.32.0; http://www.bioconductor.org/packages/release/bioc/html/topGO.html). GO annotations were obtained from the GO.db package (version 3.5.0; https://bioconductor.org/packages/3.6/data/annotation/html/GO.db.html), using the release dated 2017-11-01. GO enrichment analysis was performed to explore the functions of genes producing these dysregulated expressed circRNAs. The criterion for determining significant function was set at *P* < 0.05.

### Identification strategy for conserved mouse-human circRNAs

To identify evolutionarily conserved circRNAs between mice and humans, we employed a multi-step screening approach. First, we identified differentially expressed circRNAs in the CI-AKI mouse model using dual criteria (fold change > 2 and *P* < 0.05) and restricted our analysis to exon-derived circRNAs ranging from 200 to 2000 nucleotides in length. We then mapped these circRNAs to their corresponding gene symbols using circBase (http://www.circbase.org/). Next, we identified human homologous genes and their associated hsa_circ_RNAs 39, selecting human circRNAs of comparable length to their mouse counterparts. Sequence conservation was assessed using BLAST alignment (https://blast.ncbi.nlm.nih.gov/Blast.cgi), with stringent criteria of > 85% sequence identity and > 95% query coverage. Finally, we validated the conservation status of the identified circRNA pairs using circBank (http://www.circbank.cn/).

### Virus infection

The *in vivo* function of *circSamd4* was investigated using an adeno-associated virus 9 (AAV9)-mediated knockdown approach. AAV9 vectors containing either *circSamd4* short hairpin RNA (shRNA) or scrambled control shRNA (AAV9-NC) (Hanbio Biotechnology Co., Ltd., Shanghai, China) was diluted to 1 × 10¹² viral genomes/mL in PBS. The *circSamd4* shRNA sequence was 5'-GCACGAGAAUCAUUAACCAAU-3'. Multiple sites in the left renal cortex were injected with either AAV9-*circSamd4* shRNA or AAV9-NC using an insulin syringe. The mice were subjected to CI-AKI induction after one week post injection.

### Generation of tubule-specific *Cirbp* knockout mice

*Cirbp* homozygous floxed (*Cirbp^fl/fl^*) mice and *Ggt1-Cre* mice (both C57BL/6 background) were provided by Cyagen Biosciences Inc. (Suzhou, China). *Cirbp^fl/fl^* mice were crossed with *Ggt1-Cre* mice to produce tubule-specific *Cirbp* knockout mice (*Ggt1-Cre^+^/Cirbp^fl/fl^*). Their *Ggt1-Cre^-^/Cirbp^fl/fl^* littermates served as controls. Genotyping was performed by PCR analysis of tail-derived genomic DNA (gDNA) using a Mouse Direct PCR Kit (Bimake, Houston, TX). All PCR primer sequences are provided in Table S1.

### Plasmid construction

The *circSAMD4* and CIRBP overexpression plasmids were generated using pCD25-ciR vector (Geneseed, Guangzhou, China) and CV702 vector (Genechem, Shanghai, China), respectively. Additionally, the truncated CIRBP (Δ1-90) and truncated CIRBP (Δ91-172) overexpression plasmids were also generated using CV702 vector (Genechem, Shanghai, China).

### Cell culture and treatments

The immortalized proximal RTEC line (HK-2 cells) initially from ATCC and 293T cells were maintained in DMEM/F-12 and DMEM respectively, both containing 10% FBS and 1% antibiotics (Gibco, Carlsbad, CA). Based on our previous study 40, HK-2 cells were exposed to 200 mg iodine/mL iohexol for 6 h.

For loss-of-function studies, HK-2 cells were transfected with *circSAMD4* small interfering RNA (siRNA) (Hanbio Biotechnology, Shanghai, China), *SRSF3* siRNA, or *CIRBP* siRNA (Tsingke Biotechnology, Beijing, China), or negative control using Lipofectamine 2000 (Invitrogen, Carlsbad, CA) as instructed by the manufacturer. The sequences of all siRNAs used are detailed in Table S2.

For gain-of-function experiments, all plasmid transfections, including corresponding empty vectors, were conducted with Lipofectamine 3000 (Invitrogen, Carlsbad, CA) as recommended by the manufacturer.

### Isolation and culture of human primary RTECs

Human primary RTECs were harvested from the normal renal cortical tissue located at a minimum distance of 4 cm from tumor margins. The cortical tissue was mechanically dissected and enzymatically digested using Hanks' balanced salt solution containing collagenase II (1 mg/mL; Sigma-Aldrich, St. Louis, MO). Subsequently, three kinds of sieves with mesh diameters of 100 μm, 70 μm, and 40 μm were used to filter the mixture in turn. The final 40 μm filtrate was collected and centrifuged. The cell pellet was seeded into culture dishes. RTECs were confirmed by immunofluorescence staining for Cytokeratin 18 (A01357-1, BOSTER, Wuhan, China). For some experiments, human primary RTECs were stimulated by 200 mg iodine/mL iohexol for 6 h.

### RNA Extraction and qRT-PCR

Total RNAs from kidney tissues and cells was extracted with TRIzol reagents (TaKaRa, Dalian, China). Next, RNA was reversely transcribed to complementary DNA (cDNA) utilizing PrimeScript™ RT reagent Kit (TaKaRa, Dalian, China). qRT-PCR was performed using the TB Green Premix Ex Taq™ II (TaKaRa, Dalian, China) on a LightCycler 96 Real-Time PCR System. All primer sequences are provided in Table S1. Gene expression was determined using primer amplification curves, melting curves, and CT values.

### RNA Fluorescence *in Situ* Hybridization (FISH) analysis

The Ribo™ FISH kit (Ribobio, Guangzhou, China) was used to perform RNA-FISH experiments on the basis of the instructions in test kit. The specific probes spanning the splice junction site of *circSAMD4* or *circSamd4* were labelled with Cy3 and synthesized by RiboBio, which also provided the Cy3-labeled *U6* and *18S* probes. For *in vitro* experiments, HK-2 cells or human primary RTECs grown on slides were fixed, permeabilized, and pre-hybridized. The cells were then incubated with hybridization buffer containing specific probes overnight at 37 °C under dark conditions, followed by washing with hybridization washing solutions and PBS. Nuclei were counterstained with DAPI. For *in vivo* FISH, paraffin-embedded mouse or human kidney sections were deparaffinized, hydrated, and permeabilized with proteinase K (GenePharma, Shanghai, China) prior to hybridization. Staining intensity was quantified using a blinded approach. From each human specimen, six random microscopic fields at 20× magnification were selected for quantification of *circSAMD4*-positive regions using ImageJ software.

### RNase R treatment

RNA samples extracted from HK-2 cells or human primary RTECs were incubated with 3 U/μg RNase R (Geneseed, Guangzhou, China) at 37 °C for 30 min 41. The expression levels of *circSAMD4*, linear *SAMD4A*, and *GAPDH* were then analyzed by qRT-PCR following RNase R digestion.

### TUNEL staining

Cell death in kidney after CI-AKI was evaluated utilizing by TUNEL assay (Roche Diagnostics, Basel, Switzerland). In brief, after deparaffinization and permeabilization with 0.3% Triton X-100, kidney sections were stained with the TUNEL reaction assay at 37 °C for 1 h under dark conditions. The sections were counterstained with DAPI and imaged under fluorescence microscopy. Ten random fields per section were analyzed to quantify TUNEL-positive cells.

### Immunoblot analysis

Renal tissues or cells were lysed by RIPA buffer mixed with 1% protease inhibitor (cwbio, Beijing, China). Equal amounts of protein were separated by sodium dodecyl sulfate polyacrylamide gel and transferred to polyvinylidene fluoride membrane. After blocking with 5% bovine serum albumin (BSA) for 1 h, membranes were then incubated with primary antibodies overnight at 4 °C, followed by corresponding secondary antibodies for 1 h at room temperature. The specific primary antibodies described below were used: cleaved caspase-3 (9664, Cell Signaling Technology, Danvers, MA), SRSF3 (ab198291, Abcam, Cambridge, UK), CIRBP (ab191885 or ab246510, Abcam, Cambridge, UK), Fas (4233, Cell Signaling Technology, Danvers, MA) and GAPDH (10494-1-AP, Proteintech, Wuhan, China). The second antibodies were purchased from Servicebio (GB23303, Wuhan, China).

### Flow cytometry analysis

Annexin V - Alexa Fluor 647 / propidium iodide (PI) Apoptosis detection Kit was purchased from 4 A Biotech (Beijing, China) to evaluate cell apoptosis. Concisely, cells were collected using 0.25% EDTA-free trypsin and washed with cold PBS. Following a 10-min incubation with Annexin V (5 μL) at room temperature, PI solution (10 μL, 20 μg/mL) was added. Flow cytometric analysis was conducted within 1 h, with subsequent data processing using FlowJo software. Apoptotic cells were calculated as the sum of Annexin V single-positive (Q3) and Annexin V/PI double-positive (Q2) populations.

### RNA immunoprecipitation (RIP) assay

The RIP assay was conducted with a commercial kit (bes5101, BersinBio, Guangzhou, China). Concisely, the cells were collected, lysed, and then reacted with an anti-IgG antibody, or anti-SRSF3 antibody (RN080PW, MBL, Tokyo, Japan), or anti-FLAG antibody (F1804, Sigma-Aldrich, USA) at 4 °C for 16 h. The immune complexes were then captured with Protein A/G magnetic beads at 4 °C for 1 h and washed with polysome washing buffer. RNA was eluted by incubating the samples with polysome elution buffer at 55 °C for 1 h. The eluted RNA was purified using TRIzol Reagent and analyzed by qRT-PCR as described above.

### Immunofluorescence staining

Cells were washed with PBS, then fixed in 4% paraformaldehyde for 5 min at room temperature, followed by permeabilization with pre-cooled methanol for 10 min at -20 °C. After a 1-h blocking step at room temperature, cells were incubated overnight at 4 °C with primary antibodies against CIRBP (10209-2-AP, Proteintech, Wuhan, China) or Cytokeratin 18 (A01357-1, Boster, Wuhan, China). The cells were then incubated with secondary antibodies - either Alexa-Fluor 488 (ab150077, Abcam, Cambridge, UK) or CoraLite594 (SA00013-4, Proteintech, Wuhan, China) - for 1 h at 37 °C. Nuclei were counterstained with DAPI (SouthernBiotech, Birmingham, AL). For quantification, nuclear and cytoplasmic fluorescence intensities were measured in individual cell using ImageJ software, and their ratio was calculated.

For *in vivo* analyses, kidney sections (4 µm) were deparaffinized, rehydrated through graded alcohols, and subjected to citrate-based antigen retrieval. After permeabilization and blocking with 5% BSA, sections were incubated with primary antibodies against CIRBP (10209-2-AP, Proteintech, Wuhan, China) or SRSF3 (RN080PW, MBL, Tokyo, Japan) overnight at 4 °C, followed by secondary antibody and DAPI staining. Ten random fields per section were imaged, and ImageJ software was employed to calculate relative fluorescence intensity.

### RNA FISH- immunofluorescence examination

*In vitro*: cells were hybridized with Cy3-labeled probes specific for *circSAMD4*, washed with hybridization buffer and PBS, and then blocked for 1 hour at room temperature. Cells were incubated overnight at 4 °C with a primary antibody against CIRBP (10209-2-AP, Proteintech, Chicago, USA), followed by incubation with an Alexa Fluor 488-conjugated secondary antibody for 1 hour at 37 °C. Nuclei were counterstained with DAPI, and images were captured using fluorescence microscopy.

*In vivo*: kidney sections were blocked for 1 hour at room temperature and then incubated with fluorescein-conjugated markers for 2 hours at room temperature, including anti-lotus tetragonolobus lectin (LTL) (FL-1321), anti-peanut agglutinin (PNA) (FL-1071), and anti-dolichos biflorus agglutinin (DBA) (FL-1031) (all from VECTOR Laboratories, CA, USA). Nuclei were counterstained with DAPI, and images were acquired using fluorescence microscopy.

### Nuclear and cytoplasmic protein extraction

Nuclear and cytoplasmic proteins were isolated from HK-2 cells using the Nuclear and Cytoplasmic Protein Extraction Kit (Beyotime, Shanghai, China). Briefly, adherent cells were harvested through PBS washing, scraping, and centrifugation. Cytoplasmic protein extraction involved sequential treatment with phenylmethanesulfonyl fluoride -containing reagents A and B: samples were subjected to vigorous vortex after adding reagent A and then incubated on ice for 10-15 min. Subsequently, after adding reagent B, the sample was subjected to two vigorous vortex steps for 5 s each, separated by a 1-min incubation on ice. The sample was then centrifuged to collect the supernatant containing cytoplasmic proteins. Nuclear proteins were isolated by adding nuclear protein extraction reagent, subjecting the sample to repeated vortex and ice-bath cycles for 30 min. The nuclear protein fraction was obtained by collecting the supernatant following centrifugation.

### Immunohistochemistry analysis

Kidney biopsy sections (4 μm) were subjected to deparaffinization and gradient ethanol rehydration, followed by citrate-mediated antigen retrieval and hydrogen peroxide treatment. After being blocked with 5% BSA, the sections were then incubated overnight at 4 °C with primary antibodies against either CIRBP (10209-2-AP, Proteintech, Wuhan, China) or SRSF3 (RN080PW, MBL, Tokyo, Japan), followed by secondary antibody incubation. Immunoreactivity was visualized using diaminobenzidine (Beijing Zhongshan Jinqiao Biotechnology, Beijing, China), and sections were counterstained with hematoxylin (Servicebio, Wuhan, China). Ten random fields per section were captured for quantitative analysis, and ImageJ software was employed to measure the percentage of positive staining area.

### Patient's information

Renal biopsy specimens with biopsy-confirmed ATI were collected from the Second Xiangya Hospital, Central South University. Patient demographics and clinical data are shown in Table S3 and Table S4. Normal kidney tissues adjacent to renal cell carcinoma served as controls. The Ethics Committee of the Second Xiangya Hospital, Central South University approved this study (approval number: LYF20230056), which complied with the Declaration of Helsinki principles. The study also obtained appropriate informed consent from all participants.

### Statistical analysis

Quantitative data are presented as mean ± standard deviation (SD). Statistical analyses were performed using GraphPad Prism 8 software (San Diego, CA). Data normality was assessed using the Shapiro-Wilk test. For normally distributed data, differences between two groups were analyzed using unpaired two-tailed Student's *t*-test. Multiple group comparisons were conducted using one-way ANOVA with Tukey's multiple comparisons test or two-way ANOVA with Tukey's multiple comparisons test. Correlations between variables were evaluated using Pearson's correlation coefficient, and their relationships were further assessed by linear regression analysis. A value of *P* < 0.05 was considered statistically significant. All experiments were performed independently at least three times.

## Results

### *CircSamd4* and its human homolog *circSAMD4* were up-regulated in RTECs in CI-AKI

First, we established a CI-AKI murine model as evidenced by elevated SCr levels and distinctive tubular morphological injuries (Figure 1A-D). Then, Arraystar Mouse CircRNA Microarray analysis was performed to reveal distinct circRNA expression patterns in renal tissues between the CI-AKI group and the model control group. As shown in Figure 1E and F, the screening identified 236 significantly altered circRNA transcripts, comprising 120 upregulated and 116 downregulated transcripts in CI-AKI tissues compared to model controls. Analysis of genomic origins showed that exonic sequences accounted for 80.93% of the altered circRNAs, while sense overlapping regions contributed 14.41%. The remaining circRNAs aligned with intronic regions, intergenic regions, and antisense regions (Figure 1G). These differentially expressed circRNAs were primarily located on chromosomes 4 and 5 (Figure 1H). Next, using GO analysis, we annotated these circRNAs by examining the biological functions of their host genes. Molecular function analysis of source genes for the enhanced circRNAs revealed significant enrichment in protein binding and protein domain specific binding, suggesting that these circRNAs may influence CI-AKI pathogenesis through interactions with specific proteins (Figure 1I).

**Figure 2:**
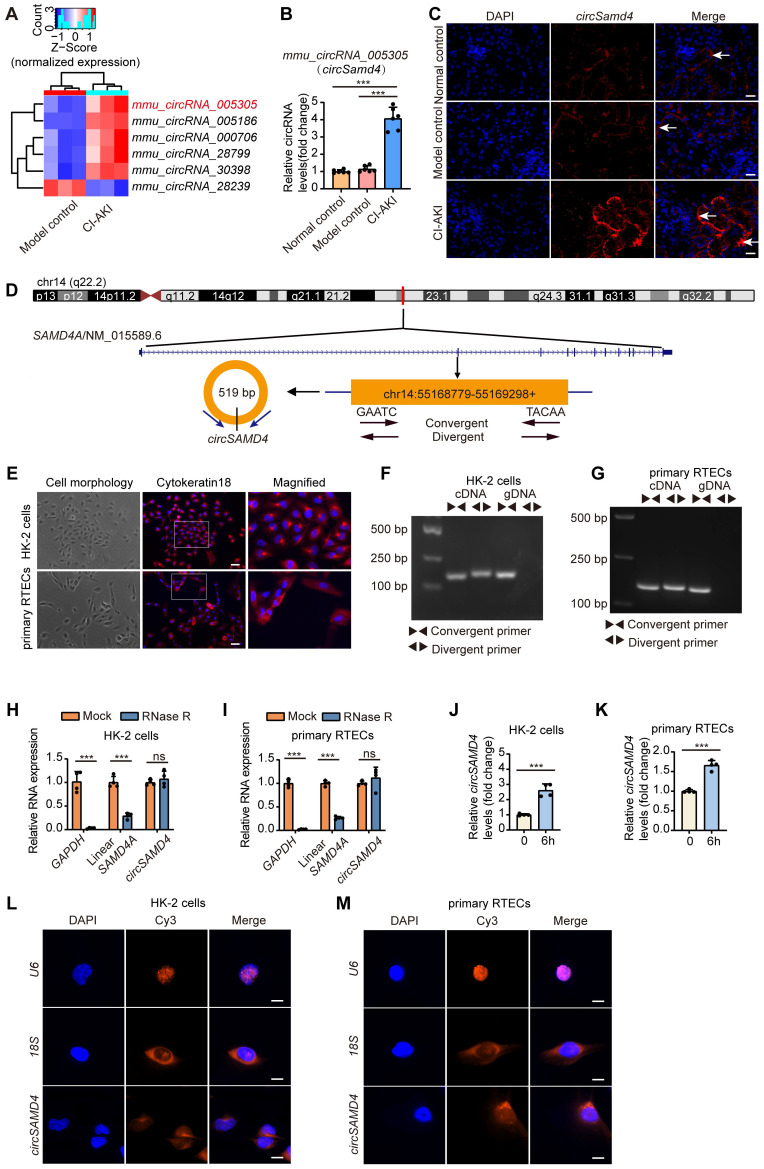
***CircSamd4* and its human homolog *circSAMD4* were up-regulated in RTECs in CI-AKI.** (A) Heatmap showing expression patterns of six conserved circRNAs (five upregulated, one downregulated). (B) C*ircSamd4* (*mmu_circ_005305*) expression in kidney tissues from normal control, model control, and CI-AKI groups, measured by qRT-PCR (n = 6). (C) RNA fluorescence in situ hybridization (FISH) analysis showing *circSamd4* expression and subcellular localization in kidney tissues after iohexol treatment. The white arrows indicated *circSamd4*-positive signals. Scale bar: 20 μm. (D) Schematic representation of *circSAMD4* genomic location and splicing pattern. *CircSAMD4* was formed by back-splicing of exon 3 of the human *SAMD4A* gene. (E) Cell morphology and representative immunofluorescence images of Cytokeratin 18 in HK-2 cells and human primary RTECs. Scale bar: 50 μm. (F and G) Agarose gel electrophoresis showing amplification of *circSAMD4* from complementary DNA (cDNA) but not from genomic DNA (gDNA) using divergent primers, while convergent primers amplified linear *SAMD4A* from both templates in HK-2 cells and human primary RTECs. (H and I) Relative abundance of *circSAMD4*, linear *SAMD4A*, and *GAPDH* in HK-2 cells and human primary RTECs with or without RNase R treatment, assessed by qRT-PCR (n = 4). (J and K) *CircSAMD4* (*hsa_circ_0004846*) expression in HK-2 cells and human primary RTECs after 6-hour iohexol (200 mg iodine/mL) exposure (n = 4). (L and M) RNA FISH analysis of *circSAMD4* subcellular localization in HK-2 cells and human primary RTECs. Nuclei were counterstained with DAPI; *U6* and *18S* served as nuclear and cytoplasmic markers, respectively. Scale bar: 10 μm. All quantitative data are presented as mean ± SD (B, and H-K). Statistical analysis was performed using one-way ANOVA with Tukey's multiple comparisons test (B) or unpaired two-tailed Student's *t*-test (H-K). ***P < 0.001.

Subsequently, we selected circRNAs (fold change > 2; nucleotide length > 200 and < 2000; exon-derived) from the microarray data. The top six circRNAs, ranked by sequence conservation identity, were shown in Table S5. Of these candidates, five circRNAs exhibited elevated expression levels, while one showed reduced abundance (Figure 2A). The characteristics of these conserved circRNAs between human and mouse were as follows: identical parental gene symbols; similar sequence lengths; sequence identity of at least 85% upon BLAST comparison; and verification of conservation in the circBank database. Among these circRNAs, we further focused on *circSamd4* (circID: *mmu_circRNA_005305*), which is derived from the mouse gene *Samd4*, and its human homolog *circSAMD4* (circID: *hsa_circ_0004846*), considering their highest identity (93%) and the highest expression abundance in renal cortical tissues (Figure S1). Consistent with the microarray data, iohexol injection significantly upregulated *circSamd4* expression in mouse kidney tissues (Figure 2B). Furthermore, FISH assay visually demonstrated the upregulation of *circSamd4* levels mainly in the cytoplasm of RTECs of CI-AKI mice (Figure 2C). To further examine the localization of circ*Samd4*, we performed co-staining with segment-specific tubular markers: LTL for proximal tubules, PNA for distal tubules, and DBA for collecting ducts. As shown in Figure S2, *circSamd4* was extensively co-localized in renal tubules, particularly in proximal tubules, with minimal expression in collecting ducts.

Then, we further explored the characteristics of *circSAMD4*. As shown in Figure 2D, *circSAMD4* was derived from exon 3 of the human *SAMD4A* gene (NM_015589.6 transcript) and was located in the 55168779-55169298 region of chromosome 14. Both HK-2 cells and human primary RTECs were identified by Cytokeratin 18 expression using immunofluorescence staining (Figure 2E). Nucleic acid electrophoresis demonstrated that *circSAMD4* was exclusively detected using divergent primers on cDNA templates, while remaining undetectable in gDNA samples. However, *SAMD4A* mRNA was successfully amplified by convergent primers regardless of template source (cDNA or gDNA) in both cell types (Figure 2F and G). Additionally, the circular configuration of *circSAMD4* was further validated by its stability during RNase R treatment, unlike the significant degradation observed for both *GAPDH* and linear *SAMD4A* transcripts exposed to the same enzyme (Figure 2H and I). Exposure of HK-2 cells to iohexol for 6 h significantly increased *circSAMD4* expression, a trend that was also observed in iohexol-treated human primary RTECs (Figure 2J and K). FISH assays further revealed that *circSAMD4* predominantly localized to the cytoplasm of HK-2 cells and human primary RTECs (Figure 2L and M). Collectively, these findings demonstrated that both *circSamd4* and its human homolog *circSAMD4* were up-regulated in RTECs in CI-AKI.

### Inhibition of *circSamd4* in mice ameliorated CI-AKI

**Figure 3:**
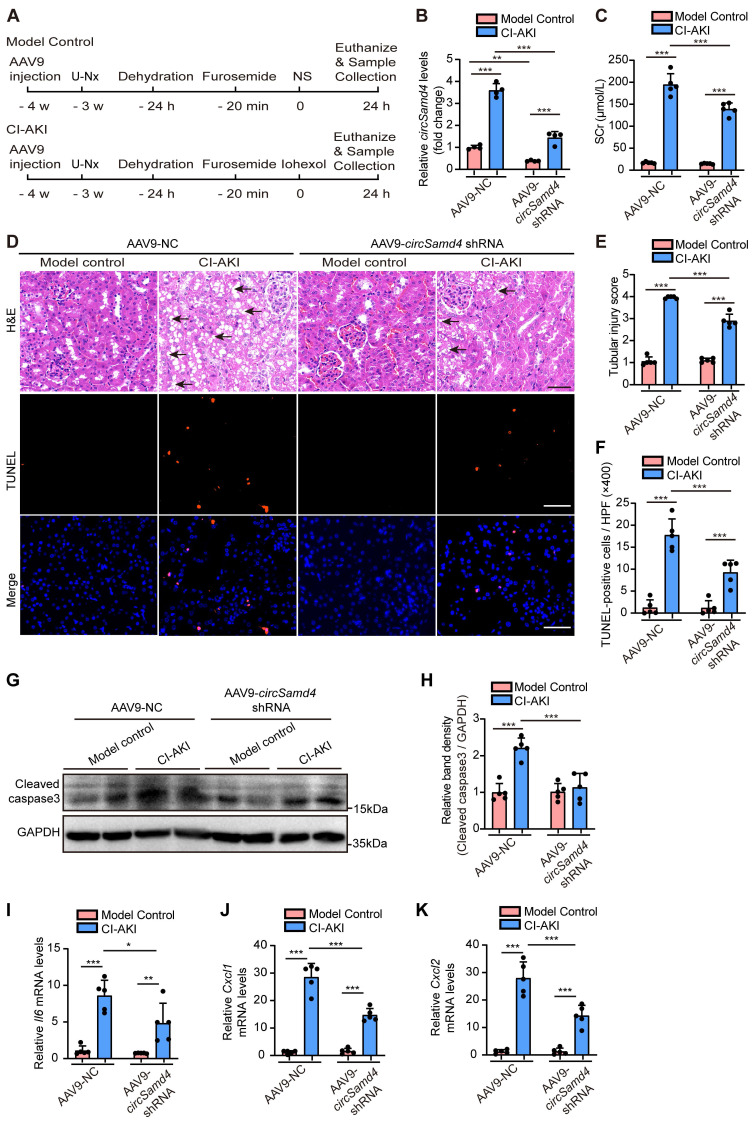
***CircSamd4* knockdown protected against CI-AKI in mice.** Male C57BL/6J mice received multi-site injections of AAV9-*circSamd4* shRNA or AAV9-NC into the left renal cortex. Subsequently, the mice were subjected to CI-AKI or model control establishment. Briefly, one week after the injection, the mice underwent unilateral nephrectomy (U-Nx) and were allowed a 3-week recovery period. The mice then received sequential interventions: 24-hour water deprivation, furosemide administration via tain vein injection (10 mL/kg), and iohexol (10 mL/kg) or normal saline (NS) injection via tail vein after 20 minutes. Serum and kidney tissues were harvested 24 hours after iohexol or NS administration. (A) Schematic illustration of the experimental design. (B) Relative *circSamd4* levels of kidney tissues detected by qRT-PCR (n = 4). (C) Serum creatinine (SCr) levels (n = 5). (D) Representative images of hematoxylin and eosin (H&E) and TUNEL staining in kidney tissues. The black arrow indicated vacuolar degeneration of tubular epithelial cells. Scale bar: 50 μm. (E) Tubular injury score in kidney tissues (n = 5). (F) Quantification of TUNEL-positive cells in kidney tissues (n = 5). (G and H) Immunoblot and densitometric analyses of cleaved caspase-3 expression in kidney cortex (n = 5). (I-K) qRT-PCR analysis of *Il6, Cxcl1, and Cxcl2* mRNA levels (n = 5). All quantitative data are presented as mean ± SD, and analyzed by two-way ANOVA with Tukey's multiple comparisons test (B, C, E, F, H-K). *P < 0.05, **P < 0.01, ***P < 0.001.

To explore the role of *circSamd4* in CI-AKI pathogenesis, we administered AAV9 carrying *circSamd4* shRNA into the left renal cortex of mice via in situ renal injection, followed by the construction of a CI-AKI model (Figure 3A). Figure 3B indicates the significant reduction of *circSamd4* in mice following local injection of AAV9-*circSamd4* shRNA into the renal cortex. AAV9-*circSamd4* shRNA did not induce renal functional or structural damage in model control mice, but it remarkably reduced SCr levels and improved renal tubular morphology in CI-AKI mice (Figure 3C-E). TUNEL assays further showed a marked decrease in cell death in renal tissues of CI-AKI mice treated with AAV9-*circSamd4* shRNA compared to those treated with AAV9-NC (Figure 3D and F). Consistently, the inhibition of *circSamd4* expression significantly reversed the accumulation of cleaved caspase-3 in kidney cortical tissues of CI-AKI mice (Figure 3G and H). Additionally, mRNA levels of the inflammatory cytokine *Il6* and chemokines *Cxcl1 and Cxcl2* were significantly elevated in the renal cortical tissues of CI-AKI mice, whereas *circSamd4* inhibition markedly reduced their expression (Figure 3I-K). Taken together, these data revealed the pathological role of *circSamd4* in CI-AKI.

### *CircSAMD4* aggravated iohexol-induced apoptosis in RTECs *in vitro*

**Figure 4:**
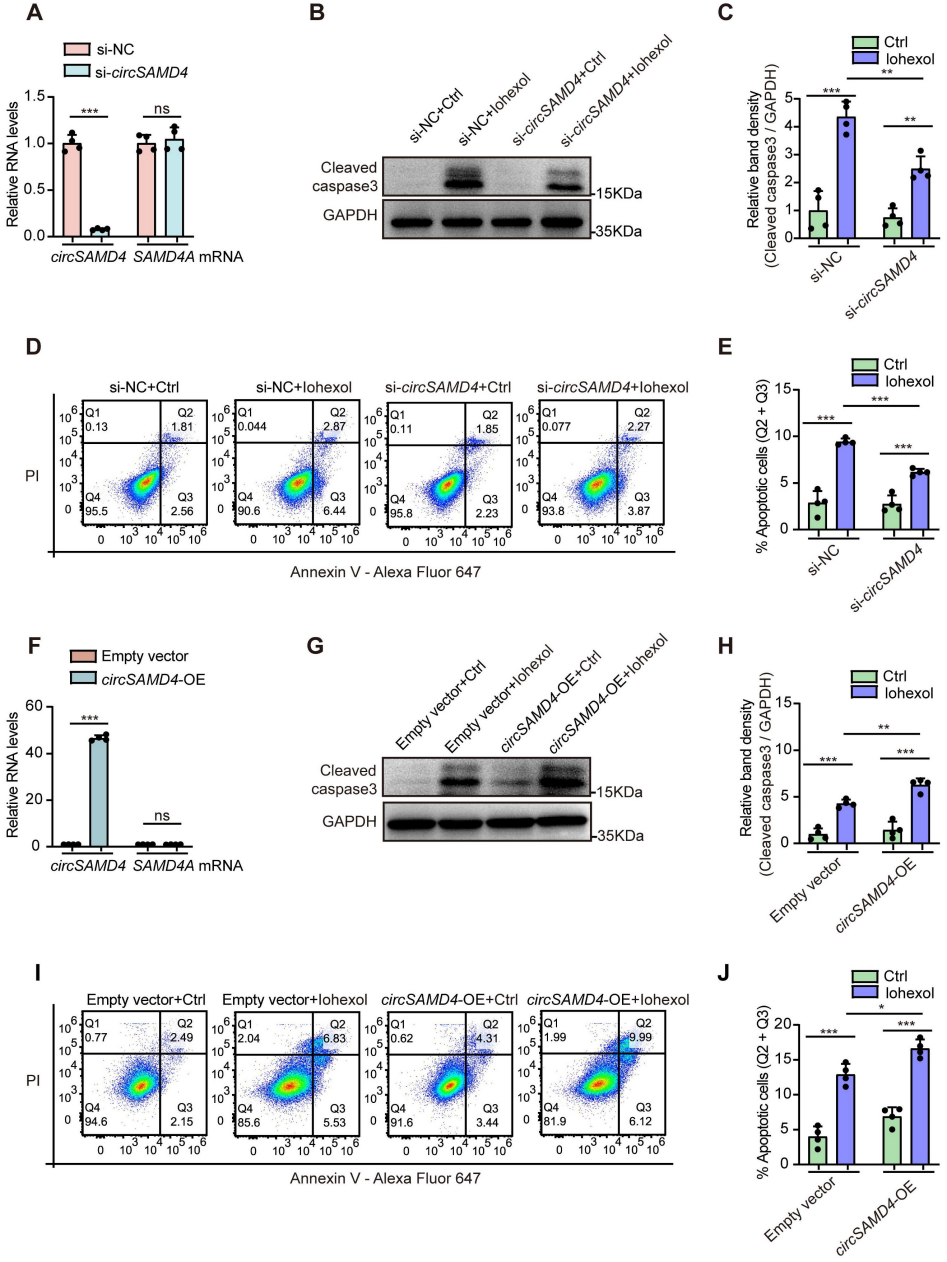
***CircSAMD4* aggravated apoptosis in HK-2 cells treated by iohexol.** (A) Relative expression of *circSAMD4* and linear *SAMD4A* in HK-2 cells transfected with *circSAMD4*-targeting siRNAs (n = 4). (B and C) Immunoblot and densitometric analyses of cleaved caspase-3 in HK-2 cells. Cells were transfected with *circSAMD4* siRNA or control siRNA for 48 hours, followed by 6-hour treatment with iohexol (200 mg iodine/mL) (n = 4). (D and E) Flow cytometric analysis of apoptosis in HK-2 cells. Cells were treated as in (B), then stained with Annexin V-Alexa Fluor 647 and PI (n = 4). (F) Relative expression of *circSAMD4* and linear *SAMD4A* in HK-2 cells transfected with *circSAMD4* overexpression plasmid (n = 4). (G and H) Immunoblot and densitometric analyses of cleaved caspase-3 in HK-2 cells. Cells were transfected with *circSAMD4* overexpression plasmid or empty vector for 48 hours, followed by 6-hour treatment with iohexol (200 mg iodine/mL) (n = 4). (I and J) Flow cytometric analysis of apoptosis in HK-2 cells. Cells were treated as in (G), then stained with Annexin V-Alexa Fluor 647 and PI (n = 4). All quantitative data are presented as mean ± SD (A, C, E, F, H, and J). Statistical analysis was performed using unpaired two-tailed Student's *t*-test (A and F) or two-way ANOVA with Tukey's multiple comparisons test (C, E, H and J). *P < 0.05, **P < 0.01, ***P < 0.001, ns, not significant.

As RTECs apoptosis exerted a vital effect on CI-AKI 35, 42, we then investigated the effect of *circSAMD4* on the apoptosis of HK-2 cells induced by iohexol. Transfection with siRNA targeting the splice junction site of *circSAMD4* effectively inhibited *circSAMD4* expression without affecting *SAMD4A* mRNA levels (Figure 4A). Exposure to iohexol markedly elevated cleaved caspase-3 levels in HK-2 cells; however, this effect was significantly attenuated following *circSAMD4* knockdown (Figure 4B and C). Consistently, flow cytometry analysis indicated silencing *circSAMD4* notably reduced the apoptosis rate of iohexol-induced HK-2 cells (Figure 4D and E).

In turn, we investigated the effects of *circSAMD4* overexpression on apoptosis of HK-2 cells stimulated by iohexol. *CircSAMD4* overexpression notably elevated *circSAMD4* levels without altering *SAMD4A* mRNA levels in HK-2 cells (Figure 4F). As we expected, immunoblot analysis demonstrated that overexpressing *circSAMD4* obviously enhanced cleaved caspase-3 levels in iohexol-treated HK-2 cells (Figure 4G and H). Similarly, flow cytometry analysis reiterated that *circSAMD4* overexpression markedly increased apoptosis rate in iohexol-stimulated HK-2 cells (Figure 4I and J). In summary, the above results consistently indicated that *circSAMD4* exacerbated apoptosis in iohexol-stimulated HK-2 cells.

### Knockdown of *circSAMD4* prevented cytoplasmic accumulation of CIRBP in RTECs during exposure to iohexol

**Figure 5:**
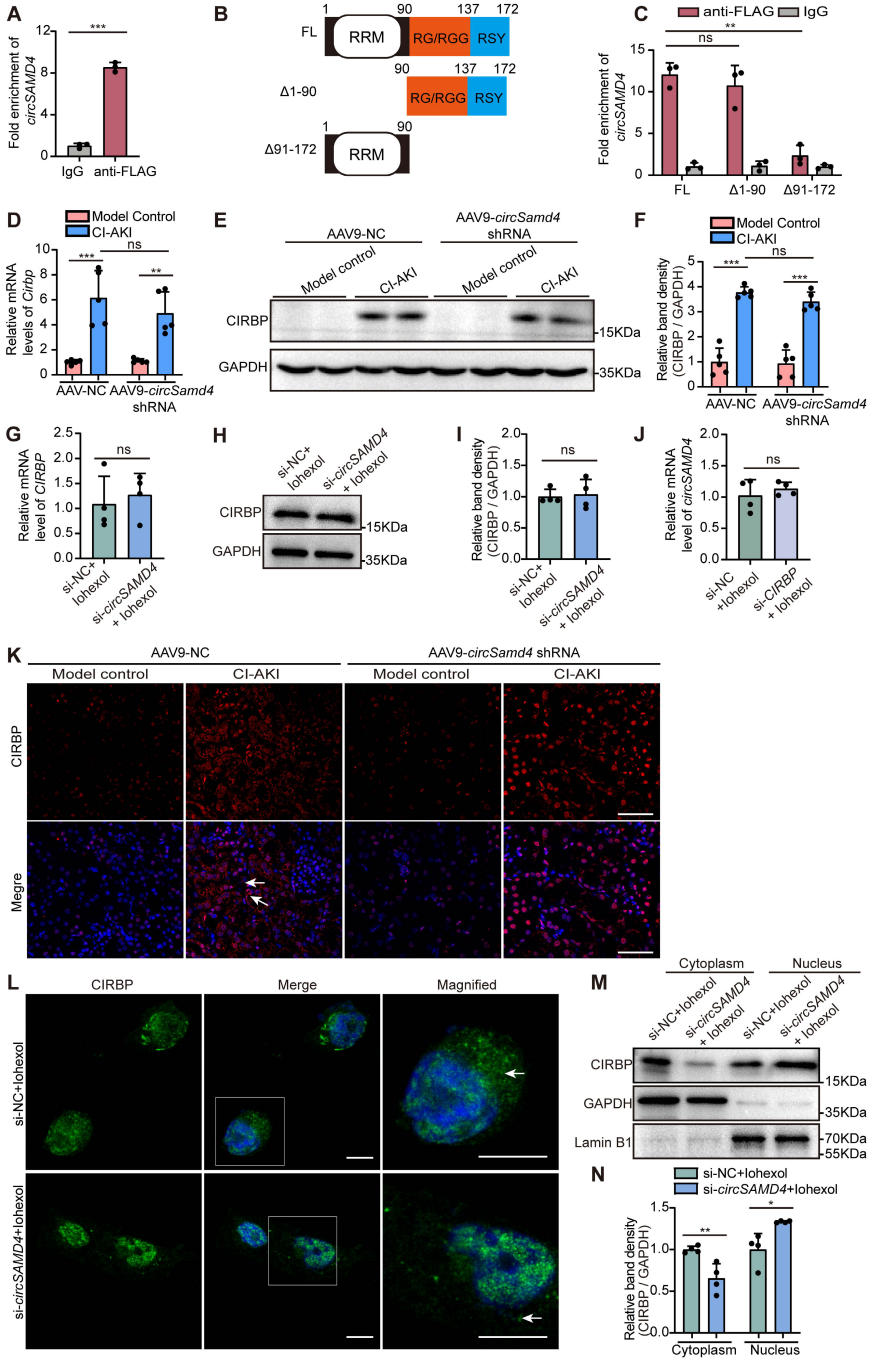
***CircSAMD4* knockdown promoted the nuclear import of CIRBP in RTECs in CI-AKI.** (A) 293T cells were co-transfected with *circSAMD4* and CIRBP-FLAG plasmids for 48 hours. The RNA immunoprecipitation (RIP) assay was performed using anti-FLAG antibody followed by qRT-PCR to detect the enrichment of *circSAMD4*. Anti-IgG antibody was served as a control (n = 3). (B) Scheme of CIRBP illustrating its domain organization and deletion of individual domains. (C) The interaction between *circSAMD4* and CIRBP was validated by RIP assay in 293T cells transfected with full length (FL) or truncated CIRBP plasmids (n = 3). (D) Relative *Cirbp* mRNA levels in kidney tissues from mice treated with AAV9-*circSamd4* shRNA and CI-AKI (n = 5). (E and F) Immunoblot and densitometric analyses of CIRBP protein in renal cortex homogenates from mice treated with AAV9-*circSamd4* shRNA and CI-AKI (n = 5). (G) Relative *CIRBP* mRNA levels analyzed by qRT-PCR in HK-2 cells. Cells were transfected with *circSAMD4* siRNA or control siRNA for 48 hours, followed by 6-hour treatment with iohexol (200 mg iodine/mL) (n = 4). (H and I) Immunoblot and densitometric analyses of whole-cell CIRBP expression in HK-2 cells treated as described in (G) (n = 4). (J) qRT-PCR analysis of *circSAMD4* expression in HK-2 cells transfected with *CIRBP* siRNA3 or control siRNA for 48 hours, followed by iohexol treatment (200 mg iodine/mL, 6 hours) (n = 4). (K) Representative immunofluorescence images of CIRBP in kidney sections from mice treated with AAV9-*circSamd4* shRNA and CI-AKI. The arrows point to the cells with cytoplasmic CIRBP. (L) Representative immunofluorescence images showing CIRBP (green) localization and nuclear DAPI staining (blue) in HK-2 cells treated as described in (G). The arrows indicate cytoplasmic CIRBP in iohexol-treated cells. Scale bar: 10 μm. (M and N) Immunoblot and densitometric analyses of nuclear and cytoplasmic CIRBP expression in HK-2 cells treated as described in (G), using GAPDH and Lamin B1 as cytoplasmic and nuclear controls, respectively (n = 4). All quantitative data are presented as mean ± SD (A, C, D, F, G, I, J, and N). Statistical analysis was performed using unpaired two-tailed Student's *t*-test (A, G, I, J, and N), one-way ANOVA with Tukey's multiple comparisons test (C) or two-way ANOVA with Tukey's multiple comparisons test (D and F). *P < 0.05, **P < 0.01, ***P < 0.001, ns, not significant.

To further elucidate the mechanisms by which *circSAMD4* contributed to CI-AKI, we sought to identify its downstream targets. A prior study identified proteins interacting with *circSAMD4* using mass spectrometry following affinity pull-down assays in both human and mouse myoblasts 41. Among them, CIRBP showed strong apoptosis regulation function in sepsis-associated or IRI-induced AKI 20. Thus, we suspected that CIRBP maybe serve as a potential downstream effector of *circSAMD4* in RTECs during CI-AKI pathogenesis. By performing RIP assay, we found that the relative enrichment of *circSAMD4* in the anti-FLAG group was significantly higher than that in the IgG group (Figure 5A). This result confirmed the interaction between *circSAMD4* and CIRBP. In addition, *circSAMD4* and CIRBP showed significant co-localization in iohexol-treated HK-2 cells (Figure S3). To investigate the underlying mechanism, we generated CIRBP variants lacking residues 1-90 or 91-172 (Figure 5B). RIP assays revealed a marked reduction in *circSAMD4* binding to the Δ91-172 variant compared with full-length CIRBP (Figure 5C), indicating that residues 91-172 are critical for the interaction. These results consistently confirm the specific interaction between *circSAMD4* and CIRBP.

Meanwhile, we found that iohexol injection significantly upregulated *Cirbp* transcript and its protein levels in the renal cortical regions of mice. However, *circSamd4* knockdown had no significant effects on *Cirbp* mRNA or protein levels in renal cortical regions from CI-AKI mice (Figure 5D-F). Similarly, inhibiting *circSAMD4* expression did not significantly alter *CIRBP* mRNA or its protein levels in iohexol-stimulated HK-2 cells (Figure 5G-I). In turn, we investigated the effect of low CIRBP levels on *circSAMD4* expression by transfection with* CIRBP* siRNA3 (Figure S4). Similarly, knockdown of *CIRBP* did not significantly affect *circSAMD4* expression in iohexol-induced HK-2 cells (Figure 5J). These results demonstrated that *circSAMD4* could not directly regulate the expression of CIRBP.

Many evidences have indicated that circRNAs can perform its function by altering the distribution of target proteins 43, 44. We then investigated the effect of *circSAMD4* on the subcellular location of CIRBP in RTECs in CI-AKI. Immunofluorescence staining showed that iohexol injection dramatically increased the cytoplasm distribution of CIRBP in RTECs of kidney tissues, but this effect was significantly suppressed by knockdown of *circSamd4* expression (Figure 5K). Consistently, immunofluorescence detection further confirmed that *circSAMD4* knockdown inhibited cytoplasmic accumulation of CIRBP in iohexol-induced HK-2 cells (Figure 5L and Figure S5). *In vitro* subcellular fractionation analysis revealed a decrease in the cytoplasm and the increase in the nucleus of CIRBP protein in iohexol-treated HK-2 cells with *circSAMD4* knockdown (Figure 5M and N). Our findings demonstrated that *circSAMD4* bound to CIRBP, and that *circSAMD4* silencing prevented CIRBP cytoplasmic accumulation in RTECs in CI-AKI.

### Renal tubule-specific *Cirbp* deletion in mice ameliorated CI-AKI

**Figure 6:**
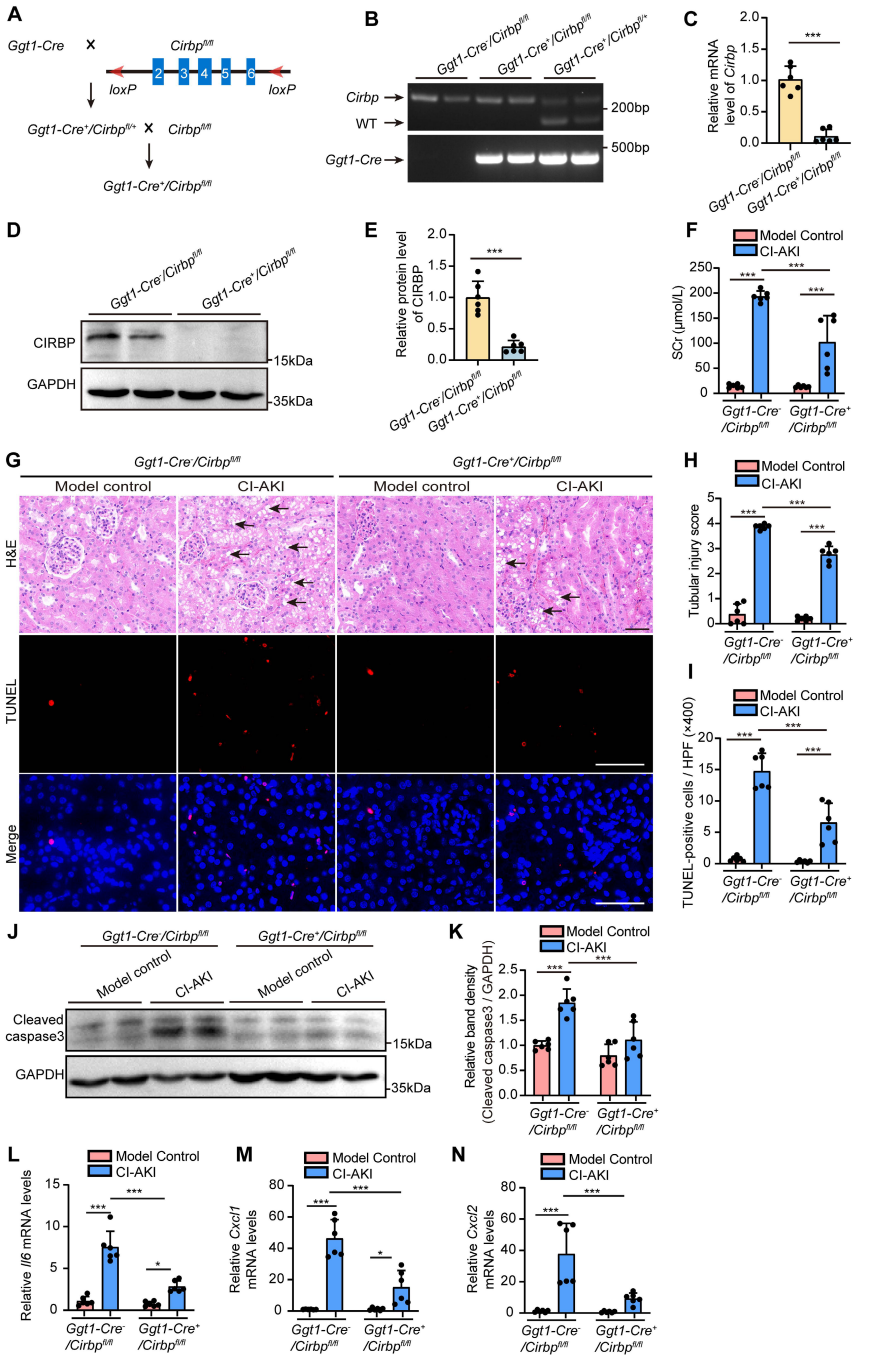
**Renal tubule-specific *Cirbp* deletion in mice ameliorated CI-AKI.** (A) The experimental scheme of generating renal tubule cell-specific *Cirbp* (*Ggt1-Cre^+^/Cirbp ^fl/fl^*) knockout mice by Cre-loxP recombinant system. (B) Genotype confirmation by PCR analysis of mouse tail DNA. (C) qRT-PCR analysis of *Cirbp* mRNA levels in renal cortex from *Ggt1-Cre^+^/Cirbp ^fl/fl^* and *Ggt1-Cre^-^/Cirbp ^fl/fl^* mice (n = 6). (D and E) Immunoblot and densitometric analyses of CIRBP expression in kidney cortex from *Ggt1-Cre^+^/Cirbp ^fl/fl^* and *Ggt1-Cre^-^/Cirbp ^fl/fl^* mice (n = 6). (F) Serum creatinine (SCr) levels from *Ggt1-Cre^+^/Cirbp ^fl/fl^* and *Ggt1-Cre^-^/Cirbp ^fl/fl^* mice with CI-AKI (n = 6). (G) Representative images of hematoxylin and eosin (H&E) staining and TUNEL staining in kidney sections from *Ggt1-Cre^+^/Cirbp ^fl/fl^* and *Ggt1-Cre^-^/Cirbp ^fl/fl^* mice with CI-AKI. The black arrow indicated vacuolar degeneration of tubular epithelial cells. Scale bar: 50 μm. (H) Tubular injury score in kidney tissues (n = 6). (I) Quantification of TUNEL-positive cells in kidney sections from *Ggt1-Cre^+^/Cirbp ^fl/fl^* and *Ggt1-Cre^-^/Cirbp ^fl/fl^* mice with CI-AKI (n = 6). (J and K) Immunoblot and densitometric analyses of cleaved caspase-3 expression in kidney cortex from *Ggt1-Cre^+^/Cirbp ^fl/fl^* and *Ggt1-Cre^-^/Cirbp ^fl/fl^* mice with CI-AKI (n = 6). (L-N) qRT-PCR analysis of *Il6, Cxcl1, and Cxcl2* mRNA levels (n = 6). All quantitative data are presented as mean ± SD (C, E, F, H, I and K-N). Statistical analysis was performed using unpaired two-tailed Student's *t*-test (C and E) or two-way ANOVA with Tukey's multiple comparisons test (F, H, I and K-N). *P < 0.05, ***P < 0.001.

In the following experiment, we established renal tubule-specific *Cirbp* knockout mice (*Ggt1-Cre^+^/Cirbp ^fl/fl^*, Figure 6A) to deeply explore the role of CIRBP in RTECs in CI-AKI. Tail genotyping, qRT-PCR assay and immunoblot analysis confirmed the successful generation of the knockout mice (Figure 6B-E). Scr levels and H&E staining clearly indicated that renal tubule-specific *Cirbp* deletion significantly improved renal function and tubular morphology in CI-AKI mice (Figure 6F-H). Consistently, TUNEL assays revealed a marked decrease in cell death in renal tissues of *Ggt1-Cre^+^/Cirbp ^fl/fl^* mice with CI-AKI compared to *Ggt1-Cre^-^/Cirbp ^fl/fl^* mice with CI-AKI (Figure 6G and I). Similarly, renal tubule-specific *Cirbp* deletion remarkably reduced accumulation of cleaved caspase-3 in renal cortical regions of CI-AKI mice (Figure 6J and K). In addition, renal tubule-specific *Cirbp* deletion significantly reduced the production of inflammatory cytokines *Il6* and chemokines *Cxcl1* and *Cxcl2* (Figure 6L-N). These results revealed CIRBP as a pathogenic mediator in RTECs during CI-AKI progression.

### Knockdown of *CIRBP* ameliorated iohexol-induced apoptosis in RTECs *in vitro*

**Figure 7:**
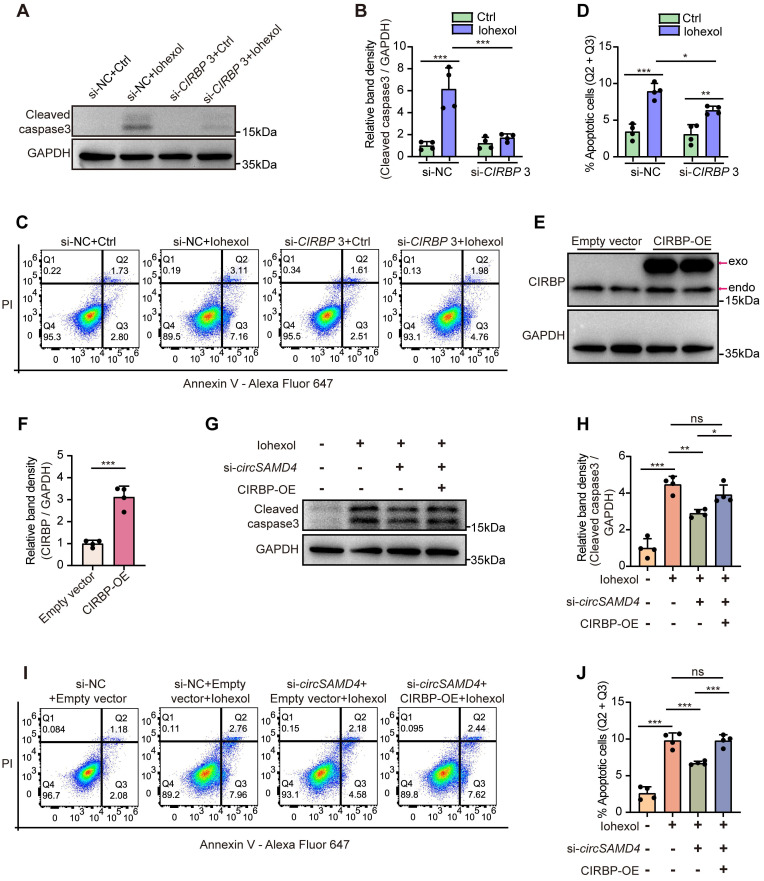
**CIRBP mediated the pro-apoptotic effect of *circSAMD4* in iohexol-treated HK-2 cells.** (A and B) Immunoblot and densitometric analyses of cleaved caspase-3 expression in HK-2 cells. Cells were transfected with *CIRBP* siRNA3 or control siRNA for 48 hours, followed by 6-hour treatment with iohexol (200 mg iodine/mL) (n = 4). (C and D) Flow cytometric analysis of apoptosis in HK-2 cells. Cells were treated as in (A), then stained with Annexin V-Alexa Fluor 647 and PI (n = 4). (E and F) Immunoblot and densitometric analyses of CIRBP expression in HK-2 cells transfected with CIRBP-FLAG plasmid or empty vector (n = 4). (G and H) Immunoblot and densitometric analyses of cleaved caspase-3 expression in HK-2 cells. Cells were co-transfected with *circSAMD4* siRNA (or control siRNA) and CIRBP-FLAG plasmid (or empty vector plasmid) for 48 hours, followed by 6-hour treatment with iohexol (200 mg iodine/mL) (n = 4). (I and J) Flow cytometric analysis of apoptosis in HK-2 cells. Cells were treated as in (G), then stained with Annexin V-Alexa Fluor 647 and PI (n = 4). All quantitative data are presented as mean ± SD (B, D, F, H and J). Statistical analysis was performed using two-way ANOVA with Tukey's multiple comparisons test (B and D), or unpaired two-tailed Student's *t*-test (F), or one-way ANOVA with Tukey's multiple comparisons test (H and J). *P < 0.05, **P < 0.01, ***P < 0.001, ns, not significant.

We next evaluated the effects of *CIRBP* knockdown on apoptosis in HK-2 cells exposed to iohexol. Inhibition of CIRBP protein level notably reduced accumulation of cleaved caspase-3 (Figure 7A and B), and decreased the apoptosis rate in iohexol-stimulated HK-2 cells (Figure 7C and D). Collectively, our findings indicated CIRBP promoted apoptosis in HK-2 cells exposed to iohexol.

### CIRBP overexpression abrogated the beneficial effects by *circSAMD4* suppression in iohexol-treated RTECs *in vitro*

Then, we assessed whether the pro-apoptotic effect of *circSAMD4* in iohexol-stimulated HK-2 cells was mediated through a CIRBP-dependent mechanism. Immunoblot analysis confirmed that the CIRBP overexpression notably elevated CIRBP protein levels in HK-2 cells (Figure 7E and F). As shown in Figure 7G and H, knockdown of *circSAMD4* could markedly decrease iohexol-caused accumulation of the cleaved caspase-3, and this effect was reversed by CIRBP overexpression. Consistently, flow cytometry analysis further verified these findings (Figure 7I and J). In summary, these results revealed that *circSAMD4* promoted apoptosis in iohexol-stimulated HK-2 cells through CIRBP-dependent mechanisms.

### CIRBP suppression attenuates apoptosis by downregulating Fas in iohexol-induced HK-2 cells

To explore the mechanism underlying the pro-apoptotic function of CIRBP, we performed RNA sequencing in iohexol-treated HK-2 cells transfected with *CIRBP* siRNA or control siRNA. Among the downregulated genes following *CIRBP* knockdown, *FAS* mRNA was significantly reduced (Figure S6A). Fas, a transmembrane protein of the TNF family, mediates inflammation and apoptosis upon binding to Fas ligand 45. *CIRBP* knockdown decreased both *FAS* mRNA and protein levels in iohexol-treated HK-2 cells (Figure S6B-D), and renal tubule-specific *Cirbp* deletion similarly reduced *Fas* mRNA in CI-AKI mouse kidneys (Figure S6E). RIP assay confirmed the binding between CIRBP and *FAS* mRNA (Figure S6F). These findings indicate that CIRBP suppression attenuates apoptosis through downregulating Fas in iohexol-induced HK-2 cells.

### SRSF3 negatively regulated *circSAMD4* production

**Figure 8:**
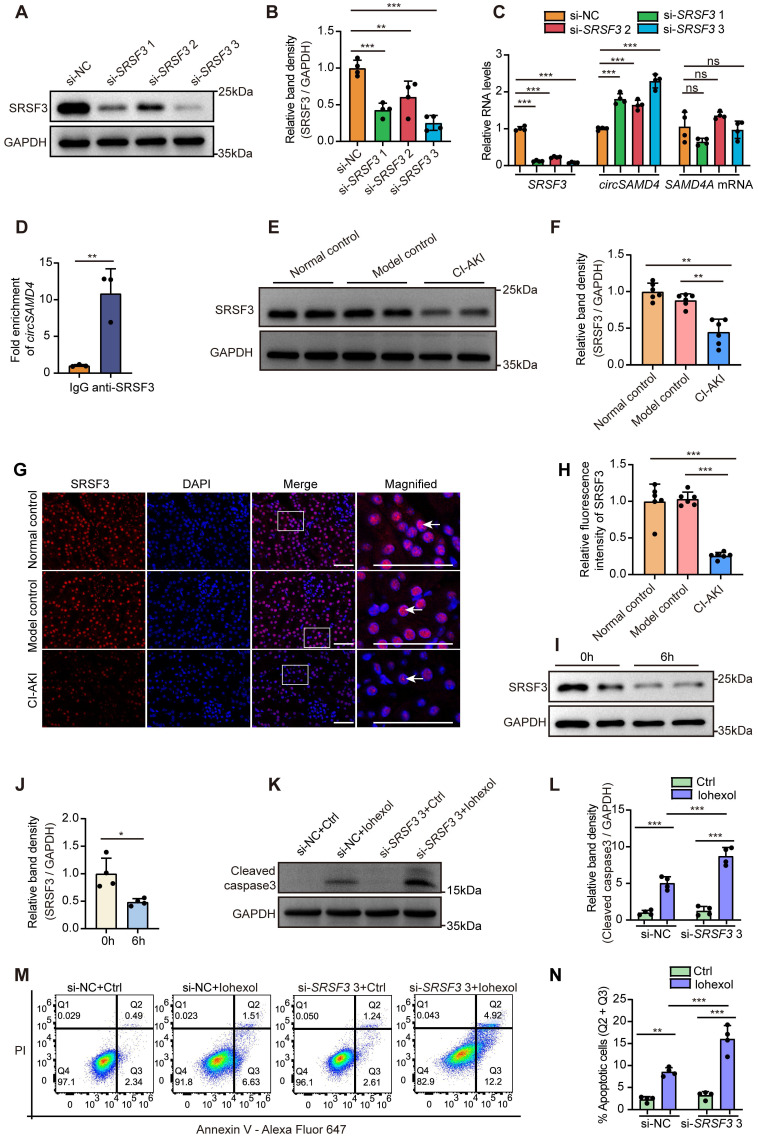
**Inhibition of SRSF3 mediated the upregulation of *circSAMD4*.** (A and B) Immunoblot and densitometric analyses of SRSF3 expression in HK-2 cells transfected with three different *SRSF3*-targeting siRNAs (n = 4). (C) Relative expression of *circSAMD4*, linear *SAMD4A* and *SRSF3* in HK-2 cells transfected with three different *SRSF3*-targeting siRNA (n = 4). (D) 293T cells were transfected with *circSAMD4* plasmid for 48 hours. The RIP assay was performed using anti-SRSF3 antibody followed by qRT-PCR to detect the enrichment of *circSAMD4*. Anti-IgG antibody was served as a control (n = 3). (E and F) Immunoblot and densitometric analyses of SRSF3 expression in kidney cortex from CI-AKI mice (n = 6). (G and H) Representative immunofluorescence images and quantification of SRSF3 staining in kidney sections from CI-AKI mice (n = 6). (I and J) Immunoblot and densitometric analyses of SRSF3 expression in HK-2 cells stimulated by iohexol for 6 hours (n = 4). (K and L) Immunoblot and densitometric analyses of cleaved caspase-3 expression in HK-2 cells. Cells were transfected with *SRSF3* siRNA3 or control siRNA for 48 hours, followed by 6-hour treatment with iohexol (200 mg iodine/mL) (n = 4). (M and N) Flow cytometric analysis of apoptosis in HK-2 cells. Cells were treated as in (K), then stained with Annexin V-Alexa Fluor 647 and PI (n = 4). All quantitative data are presented as mean ± SD (B-D, F, H, J, L and N). Statistical analysis was performed using one-way ANOVA with Tukey's multiple comparisons test (B, C, F and H), or unpaired two-tailed Student's *t*-test (D and J), or two-way ANOVA with Tukey's multiple comparisons test (L and N). *P < 0.05, **P < 0.01, ***P < 0.001, ns, not significant.

Previous studies revealed that the formation of circRNAs relied on the spliceosome machinery, including cis- and trans-regulatory elements. Reverse intronic complementary sequences facilitated spatial proximity of the 5′- and 3′-termini of an exon or of consecutive exons to induce “head-to-tail” splicing 46. However, no intronic complementary sequences existed in the flanking sequences of the back-splicing junction site of *circSAMD4*, extending 5000 bp in both upstream and downstream directions. Thus, we suspect that RNA binding proteins (RBPs) maybe closely relate to the regulation of *circSAMD4* expression. As three RBPs—SRSF3, heterogeneous nuclear ribonucleoprotein M (HNRNPM), and RNA-binding motif protein 3 (RBM3) showed the function for mediating RNA splicing 47-51, we accordingly investigated their roles in regulating *circSAMD4* formation by using siRNA knockdown assay. *SRSF3* knockdown significantly increased *circSAMD4* expression but did not obviously alter linear *SAMD4A* expression in HK-2 cells (Figure 8A-C). In contrast, silencing either *HNRNPM* or *RBM3* did not significantly affect the expression of *circSAMD4* and its linear transcript *SAMD4A* (Figure S7A and B). These results demonstrated that SRSF3 is a key regulator of *circSAMD4* formation. Moreover, bioinformatic analysis using the CATRAPID tool indicated a SRSF3 binding site in mature *circSAMD4* (Table S6). Subsequently, RIP experiments were performed to assess the interaction between SRSF3 and *circSAMD4*. The relative enrichment of *circSAMD4* in the anti-SRSF3 group was obviously higher compared to the IgG group (Figure 8D).

Next, we investigated SRSF3's expression and role in RTECs in CI-AKI. Immunoblot analysis revealed a reduction of SRSF3 expression in kidney tissues of CI-AKI mice (Figure 8E and F). Immunofluorescence detection further confirmed that iohexol remarkably decreased SRSF3 protein levels primarily in the nuclei of RTECs (Figure 8G and H). Consistently, *in vitro* exposure to iohexol markedly reduced SRSF3 protein levels in HK-2 cells (Figure 8I and J). Given that siRNA3 exhibited the most potent inhibitory effect on SRSF3 protein levels, we utilized this siRNA for subsequent functional studies. *SRSF3* knockdown significantly enhanced both cleaved caspase-3 levels (Figure 8K and L) and the apoptosis rate in iohexol-stimulated HK-2 cells (Figure 8M and N). Collectively, above data suggested that SRSF3 bound to *circSAMD4*; *SRSF3* knockdown up-regulated *circSAMD4* expression, thereby exacerbating apoptosis in iohexol-stimulated HK-2 cells.

### *CircSAMD4* knockdown abrogated the pro-apoptotic effects by SRSF3 suppression in iohexol-treated RTECs *in vitro*

Then, we assessed whether the pro-apoptotic effect of SRSF3 suppression in iohexol-stimulated HK-2 cells was mediated through a *circSAMD4*-dependent mechanism. As shown in Figure S8A and B, SRSF3 suppression could markedly increase iohexol-caused accumulation of the cleaved caspase-3, and this effect was reversed by *circSAMD4* knockdown. Consistently, flow cytometry analysis further verified these findings (Figure S8C and D). In summary, these results revealed that *circSAMD4* knockdown reverse the pro-apoptotic effects of SRSF3 depletion in iohexol-treated RTECs.

### Aberrant expression of SRSF3, *circSamd4*, and CIRBP in RTECs in cisplatin-induced AKI

To evaluate whether the dysregulation of SRSF3, *circSamd4*, and CIRBP extends beyond CI-AKI, we established a cisplatin-induced AKI mouse model (Figure S9A-C). RNA FISH and RT-PCR analyses confirmed renal accumulation of *circSamd4* (Figure S9D-E). Immunofluorescence revealed upregulation and cytoplasmic accumulation of CIRBP in RTECs, whereas SRSF3 expression was reduced (Figure S9F-G), a finding further validated by immunoblotting (Figure S9H-I). These results suggest that aberrant expression of SRSF3, *circSamd4*, and CIRBP may represent a conserved feature across different types of AKI, although additional studies are required to fully confirm this.

### Altered expression of SRSF3, *circSAMD4* and CIRBP in RTECs from human patients with biopsy-diagnosed ATI

**Figure 9:**
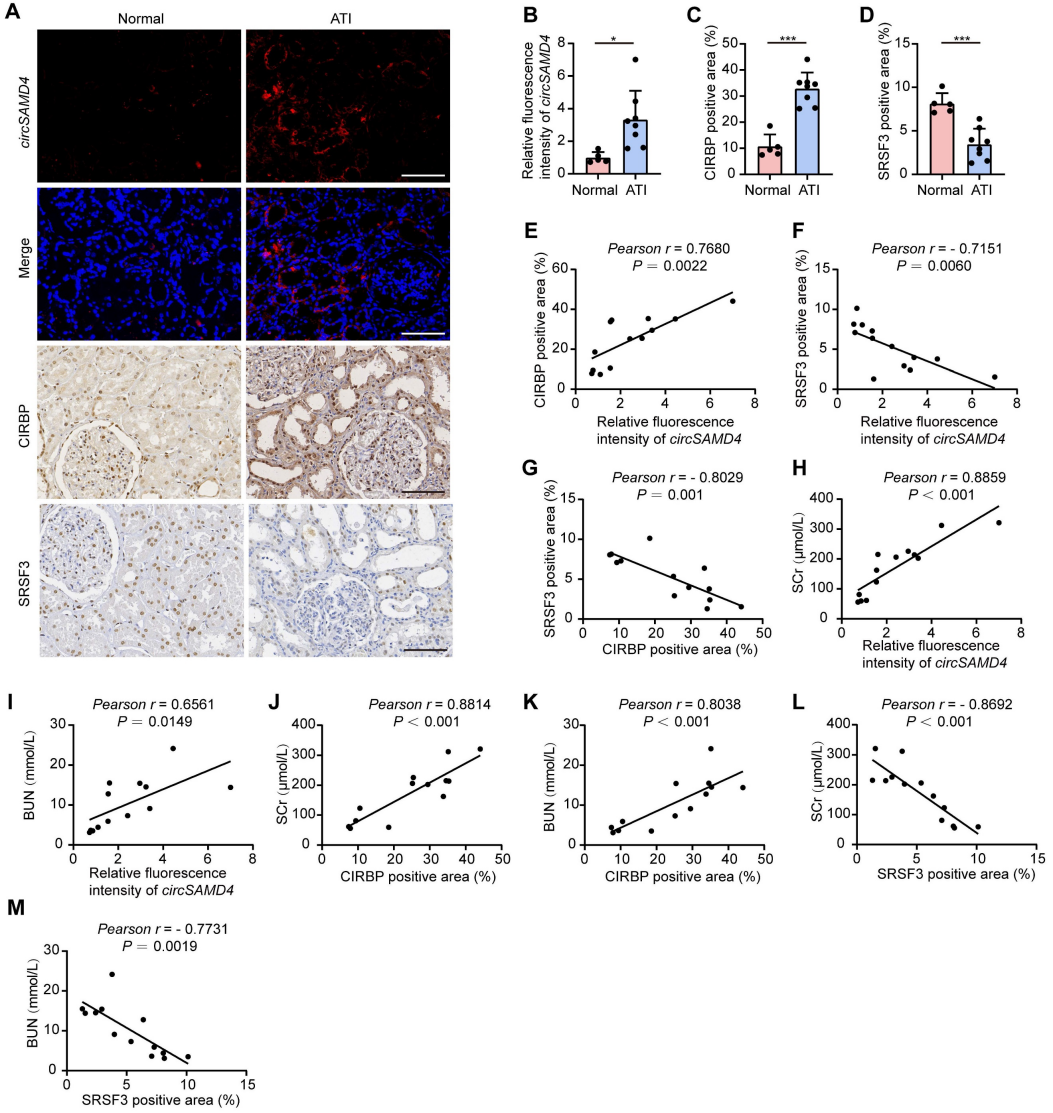
**Altered expression of SRSF3, *circSAMD4* and CIRBP in RTECs correlated with kidney injury in patients pathologically diagnosed with ATI.** (A-D) Representative RNA fluorescence in situ hybridization (FISH) staining images and quantification of *circSAMD4* expression in renal biopsy specimens from patients pathologically diagnosed with ATI (n = 8) and para-carcinoma kidney sections (n = 5). Representative immunohistochemistry images and quantitative analysis of CIRBP and SRSF3 expression from patients pathologically diagnosed with ATI (n = 8) and para-carcinoma kidney sections (n = 5). Scale bar: 100 μm. (E) Correlation between *circSAMD4* expression and CIRBP expression in all participants (n = 13). (F) Correlation between *circSAMD4* expression and SRSF3 expression in all participants (n = 13). (G) Correlation between CIRBP expression and SRSF3 expression in all participants (n = 13). (H and I) Correlation between *circSAMD4* expression and peak serum creatinine (SCr) and blood urea nitrogen (BUN) levels in all participants (n = 13). (J and K) Correlation between CIRBP expression and peak SCr and BUN levels in all participants (n = 13). (L and M) Correlation between SRSF3 expression and peak SCr and BUN levels in all participants (n = 13). All quantitative data are presented as mean ± SD (B-M). Statistical analysis was performed using unpaired two-tailed Student's *t*-test (B-D), or Pearson's correlation coefficient *r* with two-tailed *P*-value (E-M). *P < 0.05, ***P < 0.001.

To evaluate the clinical significance of SRSF3, *circSAMD4* and CIRBP in AKI, we examined their expression in kidney tissues from patients with biopsy-diagnosed ATI. RNA FISH analysis revealed a significant elevation in *circSAMD4* expression in RTECs from patients with biopsy-confirmed ATI (Figure 9A and B). We performed co-staining of *circSAMD4* with segment-specific tubular markers, including LTL for proximal tubules, PNA for distal tubules, and DBA for collecting ducts. As shown in Figure S10, *circSAMD4* was extensively co-localized in renal tubules, particularly in proximal tubules, with minimal expression in collecting ducts. Immunohistochemical staining showed upregulation and cytoplasmic accumulation of CIRBP in RTECs from patients with biopsy-confirmed ATI, whereas SRSF3 expression was reduced (Figure 9A, C and D). Additionally, *circSAMD4* expression positively correlated with CIRBP protein levels, whereas both of them negatively correlated with SRSF3 expression (Figure 9E-G). Moreover, both of the levels of *circSAMD4* and CIRBP showed positive correlation with peak SCr and blood urea nitrogen levels in patients (Figure 9H-K). Conversely, SRSF3 expression negatively correlated with kidney function in patients (Figure 9L and M).

## Discussion

Iodinated contrast media are a leading cause of AKI in hospitalized patients, yet the underlying mechanisms remain poorly understood. This study identifies the upregulation of *circSAMD4* as a pivotal driver of CI-AKI. Mechanistically, we demonstrate that *circSAMD4* inhibits nuclear import of CIRBP, while downregulation of SRSF3 promotes the expression of *circSAMD4*. Notably, depletion of *Cirbp* in RTECs significantly alleviates CI-AKI. Importantly, similar alterations in these molecules are observed in RTECs derived from patients with ATI, where their dysregulated expression correlates with the extent of kidney injury. Collectively, our findings establish the SRSF3/*circSAMD4*/CIRBP axis as a novel pathogenic pathway in CI-AKI and position *circSAMD4* as a promising therapeutic target for CI-AKI.

Recent studies have demonstrated that circRNAs play important roles in the development of AKI 11, 14, 52; however, the critical circRNAs involved in the development of CI-AKI remain unidentified. Through circRNA microarray analysis, we identified 236 differentially expressed circRNAs in kidney tissues of CI-AKI mice compared to control mice (Figure 1). As the biological properties of circRNAs become increasingly understood, their evolutionary conservation across species has garnered significant attention 6. Given that sequence conservation often implies functional relevance 53, we prioritized the six circRNAs with the highest sequence identity between mouse and human. Among these, *circSamd4* and its human homolog, *circSAMD4*, exhibited the highest sequence identity (93%). Our findings provided several evidence supporting that upregulation of *circSAMD4* contributed critically to cell death in CI-AKI: (1) *circSAMD4* was upregulated in RTECs of both murine models and cellular models of CI-AKI (Figure 2). (2) functionally, *circSamd4* knockdown in the kidney cortex mitigated renal dysfunction, tubular injury, and cell death in CI-AKI mice (Figure 3). Similar findings were observed in iohexol-treated HK-2 cells (Figure 4). (3) notably, increased *circSAMD4* expression correlated positively with kidney dysfunction in biopsy-confirmed ATI patients (Figure 9). Collectively, these findings support *circSAMD4* (or *circSamd4*) as a key contributor to CI-AKI pathogenesis, and highlight its potential as a novel clinical biomarker for CI-AKI diagnosis.

Current evidence suggests that circRNAs regulate cellular processes by interacting with proteins and modulating their nucleocytoplasmic distribution 44. In this study, we identified that *circSAMD4* interacted with CIRBP, and found that inhibiting *circSAMD4* promoted the nuclear translocation of CIRBP (Figure 5). CIRBP contains two non-classical nuclear localization signals (RG/RGG- and RSY-rich regions, residues 91-172) that are essential for nuclear import 54. The interaction with *circSAMD4* was significantly reduced in the Δ91-172 variant (Figure 5), suggesting that *circSAMD4* binds to this region to inhibit CIRBP nuclear import.

Under several cellular stress, CIRBP can relocalize to the cytoplasm, where it modulates various cellular processes, including apoptosis and proliferation 20. It is widely expressed across a variety of tissues and cell types, including those in the kidney 55. Notably, previous studies have reported conflicting roles of CIRBP in different AKI models 20. Cen et al. reported *Cirbp* knockout protected against IRI by reducing apoptosis and inflammation in mice 22, whereas another study demonstrated that CIRBP exerted renoprotective effects in AKI induced by deep hypothermic circulatory arrest 24. However, these previous studies used global *Cirbp* knockout models *in vivo*, which affected various cell types within the kidney. By using a renal proximal tubule-specific* Cirbp* depletion murine model, we demonstrated that *Cirbp* deletion in RTECs ameliorated CI-AKI in mice (Figure 6), and this finding was further supported by *in vitro* experiments (Figure 7). RNA sequencing revealed that *CIRBP* knockdown significantly reduced *FAS* mRNA, a key mediator of apoptosis and inflammation in RTECs during AKI 56-59. Consistently, CIRBP suppression decreased *FAS* mRNA and protein in iohexol-treated HK-2 cells and *in vivo*. RIP assays confirmed CIRBP binds to *FAS* mRNA (Figure S6). Collectively, our results suggest that CIRBP promotes apoptosis in CI-AKI by upregulating Fas.

SRSF3, a member of the serine/arginine-rich protein family, is involved in multiple RNA processing events, including alternative splicing 47, 60, RNA export and polyadenylation 61,62, protein translation 63,64, and pri-miRNA processing 65. In this study, we present evidence supporting the crucial role of SRSF3 in regulating *circSAMD4* expression in CI-AKI (Figure 8). Our findings revealed that *SRSF3* knockdown significantly increased *circSAMD4* expression without notably affecting *SAMD4A* mRNA levels in HK-2 cells. Furthermore, bioinformatics analysis identified a putative SRSF3 binding site within mature* circSAMD4*, and RIP assays confirmed the interaction between SRSF3 and *circSAMD4* (Figure 8). These findings align with prior research in which mass spectrometry analysis identified SRSF3 as a *circSAMD4*-associated protein following the affinity pull-down of *circSAMD4* in myoblasts 41. Similarly, it has been reported in Drosophila that SR proteins inhibit the production of Laccase2 circular RNA 26. These proteins may modulate spliceosome assembly, splicing kinetics, or circular RNA stability 26. Notably, *SRSF3* knockdown did not alter the half-life of *circSAMD4* (Figure S11), indicating that it does not affect *circSAMD4* stability. We therefore propose that SRSF3 may regulate *SAMD4A* pre-mRNA splicing via binding to flanking introns or exons. Further studies are needed to elucidate the precise mechanism.

This study also showed that *SRSF3* knockdown exacerbated iohexol-induced cell apoptosis (Figure 8), and decreased SRSF3 levels correlated with more severe kidney dysfunction in biopsy-confirmed ATI patients (Figure 9), indicating a protective role for SRSF3 in RTECs during CI-AKI. Notably, emerging evidence also supports protective effects of SRSF3 in acute injuries of other organs, including ischemic cerebral infarction and myocardial infarction 30, 31. For instance, cardiomyocyte-specific *Srsf3* deletion in mice resulted in severe systolic dysfunction and mortality within eight days 31. Collectively, the findings from others and us support an important role of SRSF3 in acute tissue injuries.

This study has direct clinical relevance by identifying the SRSF3/*circSAMD4*/CIRBP axis as a key driver of CI-AKI, linking molecular mechanisms in mouse models to patients with ATI. Dysregulation of *circSAMD4* correlates with kidney injury, and modulating *circSAMD4* or CIRBP levels can mitigate tubular cell death, highlighting potential therapeutic strategies. These findings provide a promising foundation for developing interventions to prevent or reduce CI-AKI in clinical practice, where effective treatments are currently limited.

## Conclusion

This study identifies dysregulation of the SRSF3/*circSAMD4*/CIRBP axis as a novel pathogenic mechanism in CI-AKI. Specifically, *circSAMD4* upregulation exacerbates iodinated contrast-induced injury by inhibiting nuclear import of CIRBP and triggering cell death, while SRSF3 downregulation contributes to increased *circSAMD4* expression in RTECs. These findings advance our understanding of CI-AKI pathogenesis and highlight *circSAMD4* as a promising therapeutic target and diagnostic biomarker with significant translational potential for improving CI-AKI management. Despite these compelling findings, further studies are needed to determine whether dysregulation of the SRSF3/*circSAMD4*/CIRBP axis represents a common pathogenic mechanism underlying AKI. Additionally, we confirm the dysregulation of this axis in RTECs derived from biopsy-confirmed ATI patients, with altered expression levels correlating with the severity of kidney injury. Future research should explore whether these molecules—individually or in combination—can serve as reliable biomarkers for the early diagnosis or prognosis of AKI.

## Supplementary Material

Supplementary figures and tables.

## Abbreviations

AAV9: adeno-associated virus 9
AKI: acute kidney injury
ATI: acute tubular injury
BSA: bovine serum albumin
CI-AKI: contrast-induced acute kidney injury
cDNA: complementary DNA
CIRBP: cold-inducible RNA-binding protein
circRNAs: circular RNAs
DBA: dolichos biflorus agglutinin
FISH: fluorescence in situ hybridization
gDNA: genomic DNA
GO: gene ontology
H&E: hematoxylin and eosin
HNRNPM: heterogeneous nuclear ribonucleoprotein M
IRI: ischemia-reperfusion injury
PI: propidium iodide
PNA: peanut agglutinin
RBM3: RNA binding motif protein 3
RBP: RNA binding protein
RIP: RNA immunoprecipitation
RTECs: renal tubular epithelial cells
SAMD4A: sterile alpha motif domain-containing protein 4A
SCr: serum creatinine
shRNA: short hairpin RNA
siRNA: small interfering RNA
SRSF3: serine-rich splicing factor 3
LTL: lotus tetragonolobus lectin
