# Supplementary material for: Dysregulation of SRSF3/circSAMD4/CIRBP Axis Promotes Iodinated Contrast-induced Acute Kidney Injury
Source: Int J Biol Sci. 2025 Sep 29;21(14):6156–78. doi: 10.7150/ijbs.117838 (PMC12594570; doi:10.7150/ijbs.117838)
Supplement: Supplementary file 1 — Supplementary figures and tables. [file ijbsv21p6156s1.pdf]

**Table S1. The primer sequences of targeted genes used for qRT-PCR.**

| Name | Forward Primer (5'-3') | Reverse Primer (5'-3') |
|---|---|---|
| *circSAMD4* | CCAGCACAAGTACAAGAATCATTA | AGAGTGAGCCAGGATTTTGGG |
| *circSamd4* | GCAAGCACGAGAATCATTAACCA | TTCGATAGAGTGTGCCAGGAT |
| *mmu_circRNA_005186* | GAGGATGCCTGCCGAGTTGT | CTTTGCCATAGGGGTGCGTG |
| *mmu_circRNA_000706* | CGACTAGCAACAGGGAGTGATG | CAGCAATAGCAGGGTTACCAAT |
| *mmu_circRNA_30398* | ATACTCAAACCCAAACGAAGG | CATTGTTACTGGTGCCCTCA |
| *mmu_circ_28799* | ACAAGGGCAAGAAGAGGCAC | GTGACTTGCGGCCAGGA |
| *mmu_circRNA_28239* | GCTCAATGAGGAGGATTACTGG | AAGCGTAGTCTCCACTGGTCAT |
| *SAMD4A* | TCGAGGCTTTGGGCAATCC | GAGCTGACGAATCCACTGGT |
| *SRSF3* | ATGCATCGTGATTCCTG | CTGCGACGAGGTGGAGG |
| *GAPDH* | CTGGGCTACACTGAGCACC | AAGTGGTCGTTGAGGGCAATG |
| *Gapdh* | CACTGAGCAAGAGAGGCCCTAT | GCAGCGAACTTTATTGATGGTATT |
| *CIRBP* | TTTGGGTTTGTCACCTTTG | CTGCCTGGTCTACTCGGAT |
| *Cirbp* | GGACTCAGCTTCGACACCAAC | ATGGCGTCCTTAGCGTCATC |
| *Cirbp$^{fl/fl}$* | TTTTGGATTCTGTTCCTTTGCCTC | CTTCAAGTGGGGTTTCTTTCACAC |
| *Ggt1-Cre* | CATCACATCAGGCACCCCAGAA | GAACATCTTCAGGTTCTGCGGGA |

SAMD4A: sterile alpha motif domain-containing protein 4A; SRSF3: serine-rich splicing factor 3; CIRBP: cold-inducible RNA-binding protein

**Table S2. The siRNA sequences of targeted genes used in this study.**

| Name | Sequences |
|---|---|
| si-NC | UUCUCCGAACGUGUCACGUdTdT |
| si-*circSAMD4* | AGCACAAGUACAAGAAUCAUUdTdT |
| si-*CIRBP* 1 | CUUCUCAAAGUACGGACAGAU |
| si-*CIRBP* 2 | GCCAUGAAUGGGAAGUCUGUA |
| si-*CIRBP* 3 | CCUACAGAGACAGUUAUGATT |
| si-*SRSF3* 1 | CCUGUCCAUUGGACUGUAATT |
| si-*SRSF3* 2 | UGGAACUGUCGAAUGGUGAAA |
| si-*SRSF3* 3 | CCCUCGAGAUGAUUAUCGUTT |
| si-*HNRNPM* 1 | CUGUGCAAGCUAUAUCUAUGU |
| si-*HNRNPM* 2 | ACAAGCAUAGUCUGAGCGGAA |
| si-*RBM3* 1 | AGUGGCAGGUAUUAUGACAGU |
| si-*RBM3* 2 | GGACGUUCCAGAGACUAUATT |

CIRBP: cold-inducible RNA-binding protein; SRSF3: serine-rich splicing factor 3; HNRNPM: heterogeneous nuclear ribonucleoprotein M; RBM3: RNA binding motif protein 3

**Table S3. Clinical characteristics in the normal control subjects.**

| Number | Age (year) | Sex | SCr (μmol/L) | BUN (mmol/L) |
|---|---|---|---|---|
| 1 | 56 | Female | 59.4 | 3.52 |
| 2 | 50 | Female | 55.9 | 3.11 |
| 3 | 71 | Male | 123.3 | 5.9 |
| 4 | 56 | Male | 81 | 3.64 |
| 5 | 60 | Female | 61.5 | 4.41 |

SCr: Serum creatinine; BUN: blood urea nitrogen

**Table S4. Clinical characteristics in the subjects with ATI.**

| Number | Age (year) | Sex | SCr (μmol/L) | BUN (mmol/L) | Diagnosis |
|---|---|---|---|---|---|
| 1 | 54 | Male | 321.1 | 14.4 | Nephrotic syndrome; ATI |
| 2 | 37 | Male | 312.1 | 24.16 | Nephrotic syndrome; ATI |
| 3 | 42 | Male | 225.7 | 15.44 | Nephrotic syndrome; ATI |
| 4 | 52 | Male | 215 | 15.5 | Nephrotic syndrome; ATI |
| 5 | 67 | Male | 213.7 | 14.54 | Nephrotic syndrome; ATI |
| 6 | 32 | Female | 206 | 7.3 | Henoch-Schönlein nephritis; ATI |
| 7 | 19 | Male | 202.6 | 9.11 | Nephrotic syndrome; ATI |
| 8 | 17 | Male | 162.5 | 12.78 | Nephrotic syndrome; ATI |

SCr: Serum creatinine; BUN: blood urea nitrogen; ATI: acute tubular injury

**Table S5. Top six circRNAs ranked by sequence conservation between human and mouse species.**

| Mmu_circ_RNA | | | Homologous hsa_circ_RNA | | | Conservation [a] | | |
|---|---|---|---|---|---|---|---|---|
| Seqname | Length (nt) | Gene symbol | Seqname | Length (nt) | Gene symbol | Query Cover | Identity | circBank database |
| *mmu_circRNA_005305* | 519 | *Samd4* | *has_circ_0004846* | 519 | *SAMD4A* | 99% | 93.00% | conserved |
| *mmu_circRNA_28799* | 509 | *Myh9* | *has_circ_0004470* | 509 | *MYH9* | 99% | 92.11% | conserved |
| *mmu_circRNA_28239* | 290 | *Npr3* | *has_circ_0072107* | 290 | *NPR3* | 100% | 91.72% | conserved |
| *mmu_circRNA_000706* | 420 | *Wdr1* | *has_circ_0003550* | 420 | *WDR1* | 99% | 88.54% | conserved |
| *mmu_circRNA_005186* | 744 | *Epha2* | *has_circ_0010132* | 738 | *EPHA2* | 98% | 86.84% | conserved |
| *mmu_circRNA_30398* | 529 | *Fkbp5* | *has_circ_0001599* | 527 | *FKBP5* | 99% | 85.98% | conserved |

a Conservation metrics (query coverage and identity) were determined using NCBI BLAST (https://blast.ncbi.nlm.nih.gov/Blast.cgi). Higher identity scores indicate greater homology with human circRNAs.

**Table S6. Predicted interaction between SRSF3 protein and *circSAMD4* using the CATRAPID website.**

| Gene Symbol | RNA ID | Interaction Propensity | RNA Binding Protein Propensity | RNA-Binding Domains | RNA-Binding Motifs | RNA-Binding Motifs_IDs |
|---|---|---|---|---|---|---|
| *SRSF3* | *has_circ_0004846* (*circSAMD4*) | 11.72 | 1 | 1 | 1 | P84103\|SRSF3\|UGGAC\|26876937\|PAR-CLIP |

**Supplemental Figures**

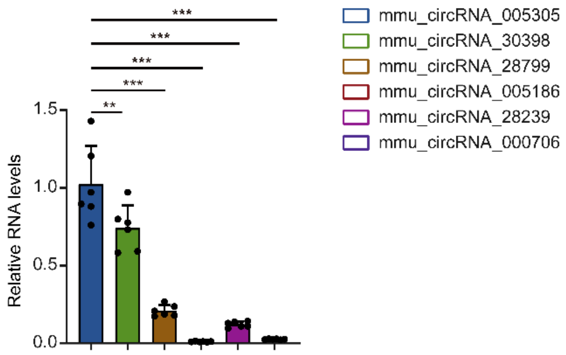


**Figure S1.** Relative circRNA levels in renal cortex tissues by qRT-PCR. All quantitative data are presented as mean ± SD. Statistical analysis was performed using one-way ANOVA with Tukey's multiple comparisons test. $^{**}P < 0.01$, $^{***}P < 0.001$.

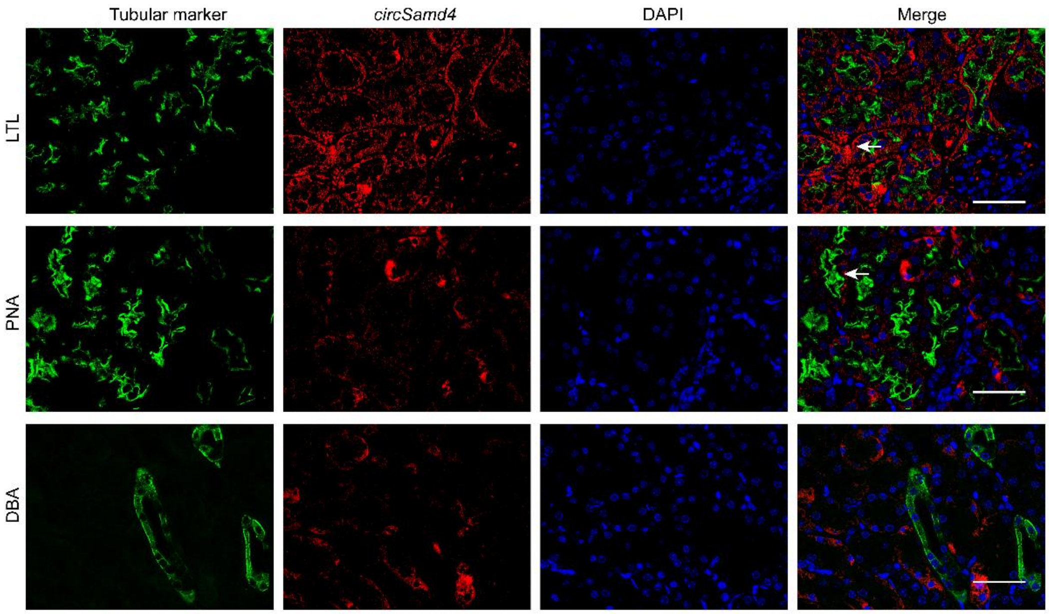


**Figure S2.** Co-localization staining of *circSamd4* and various segment-specific tubular markers in the kidneys of the CI-AKI model. *CircSamd4* (red) and various segment-specific tubular markers (green), including lotus tetragonolobus lectin (LTL), peanut agglutinin (PNA), and dolichos biflorus agglutinin (DBA), were detected by RNA FISH-immunofluorescence. Scale bar, 50 μm.

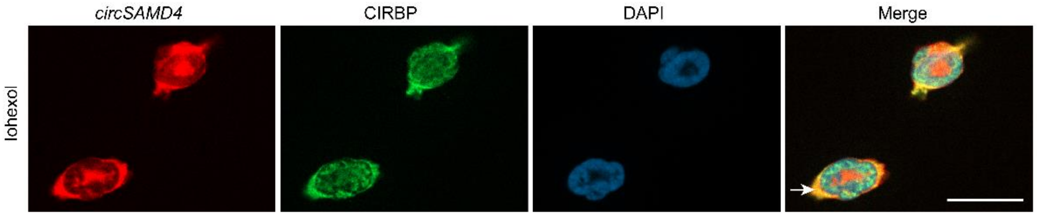


**Figure S3.** Co-localization staining of *circSAMD4* and CIRBP in iohexol-induced HK-2 cells by RNA FISH-immunofluorescence. Arrows indicate positive co-localization area. Scale bar, 20 μm.

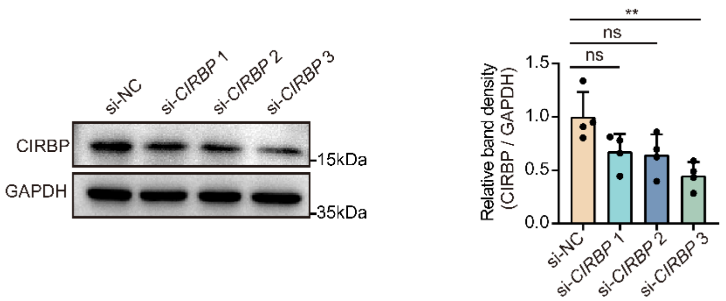


**Figure S4.** Immunoblot and densitometric analyses of CIRBP expression in HK-2 cells transfected with three different *CIRBP*-targeting siRNAs. All quantitative data are presented as mean ± SD. Statistical analysis was performed using one-way ANOVA with Tukey's multiple comparisons test (A). $^{**}P < 0.01$, ns, not significant.

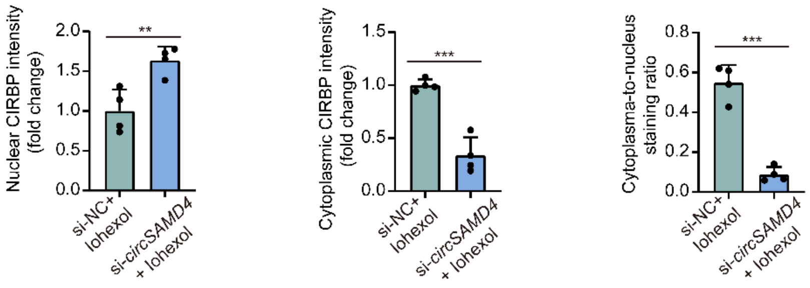


**Figure S5.** Quantification of nuclear CIRBP, cytoplasmic CIRBP, and cytoplasmic-to-nuclear CIRBP ratio, respectively (n = 4). All quantitative data are presented as mean ± SD. Statistical analysis was performed using unpaired two-tailed Student's t-test. $^{**}P < 0.01$, $^{***}P < 0.001$.

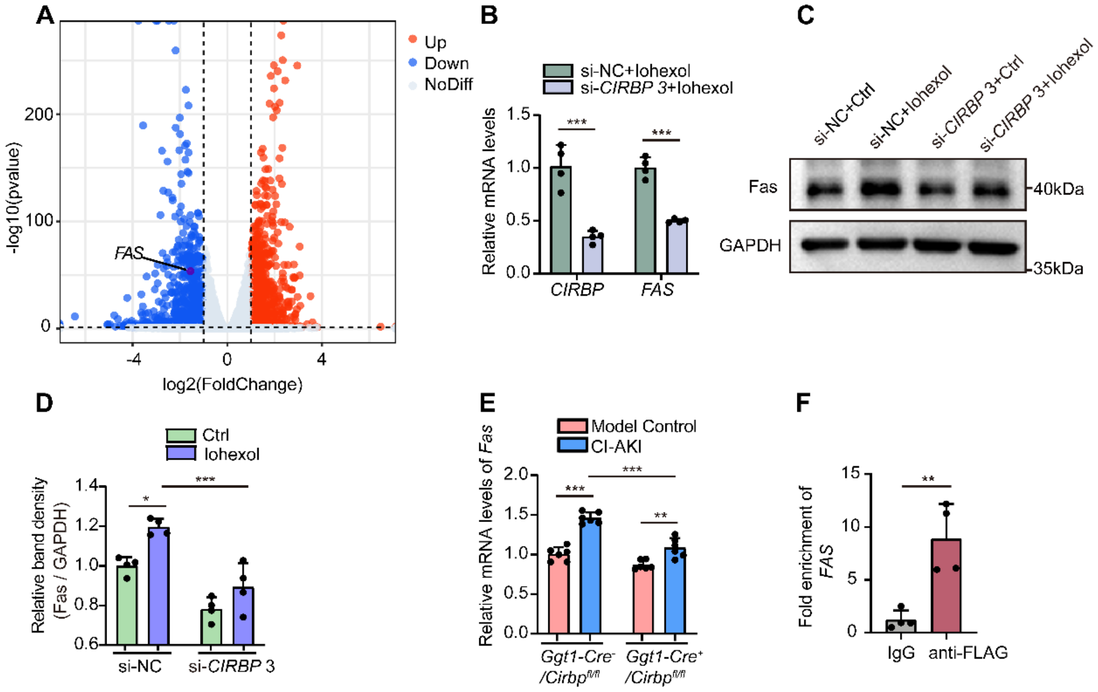


**Figure S6. CIRBP suppression attenuated apoptosis through downregulating *FAS* mRNA levels in iohexol-induced HK-2 cells.**

(A) Volcano plot of transcriptomics in iohexol-induced HK-2 cells transfected with *CIRBP* siRNA 3 or control siRNA. (B) qRT-PCR analysis of *CIRBP* and *FAS* mRNA levels in HK-2 cells transfected with *CIRBP* siRNA 3 or control siRNA for 48 hours, followed by iohexol treatment (200 mg iodine/mL, 6 hours) (n = 4). (C and D) Immunoblot and densitometric analyses of Fas in HK-2 cells. Cells were transfected with *CIRBP* siRNA 3 or control siRNA for 48 hours, followed by 6-hour treatment with iohexol (200 mg iodine/mL) (n = 4). (E) Relative *Fas* mRNA levels in kidney cortex from *Ggt1-Cre*$^{+}$*/Cirbp*$^{fl/fl}$ and *Ggt1-Cre*$^{-}$*/Cirbp*$^{fl/fl}$ mice with CI-AKI (n = 6). (F) HK-2 cells were transfected with CIRBP-FLAG plasmids for 48 hours, followed by 6-hour treatment with iohexol (200 mg iodine/mL). The RIP assay was performed using anti-FLAG antibody followed by qRT-PCR to detect the enrichment of *FAS*. Anti-IgG antibody was served as a control (n = 4). All quantitative data are

presented as mean ± SD. Statistical analysis was performed using unpaired two-tailed Student's t-test (B and F), or two-way ANOVA with Tukey’s multiple comparisons test (D and E). *$P < 0.05$, **$P < 0.01$, ***$P < 0.001$.

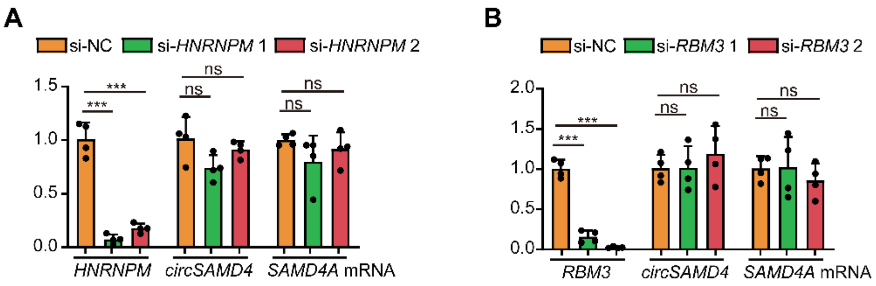


**Figure S7. Neither *HNRNPM* nor *RBM3* knockdown affected *circSAMD4* expression**

(A) Relative expression of *circSAMD4*, linear *SAMD4A* and *HNRNPM* in HK-2 cells transfected with two different *HNRNPM*-targeting siRNAs. The expression levels of these genes were normalized to *GAPDH* mRNA levels (n = 4). (B) Relative expression of *circSAMD4*, linear *SAMD4A* and *RBM3* in HK-2 cells transfected with two different *RBM3*-targeting siRNAs. The expression levels of these genes were normalized to *GAPDH* mRNA levels (n = 4). All quantitative data are presented as mean ± SD (A-B). Statistical analysis was performed using one-way ANOVA with Tukey's multiple comparisons test (A-B). ***$P < 0.001$, ns, not significant.

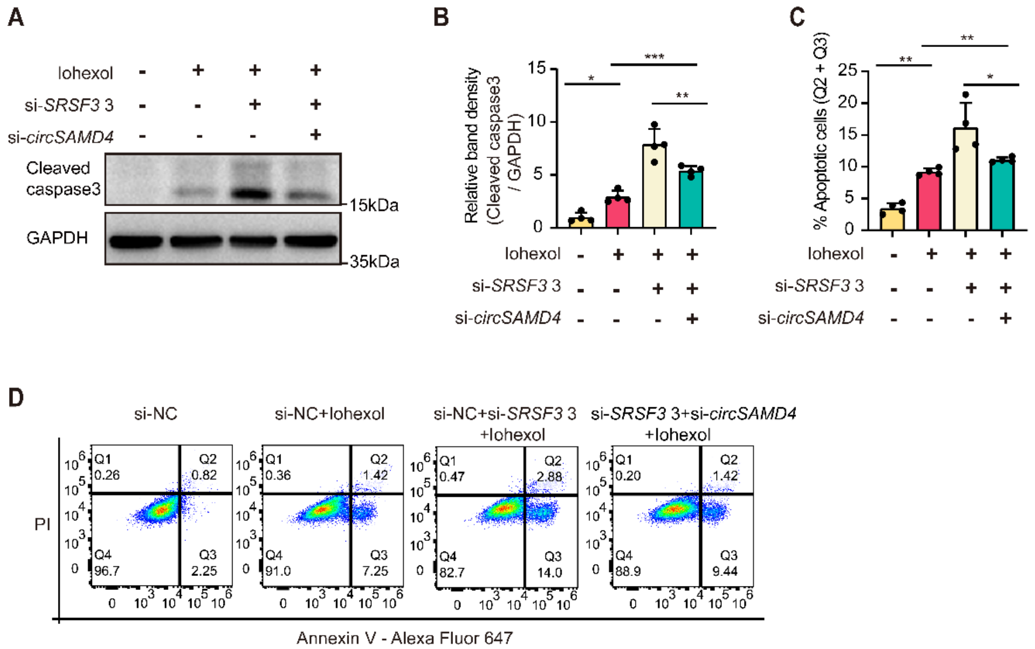


**Figure S8. *CircSAMD4* knockdown abrogated the pro-apoptotic effects by SRSF3 suppression in iohexol-treated RTECs *in vitro***

Cells were co-transfected with *SRSF3* siRNA 3 (or control siRNA) and *circSAMD4* siRNA (or control siRNA) for 48 hours, followed by 6-hour treatment with iohexol (200 mg iodine/mL) (n = 4). (A and B) Immunoblot and densitometric analyses of cleaved caspase-3 expression in HK-2 cells (n = 4). (C and D) Flow cytometric analysis of apoptosis in HK-2 cells (n = 4). All quantitative data are presented as mean ± SD (B and D). Statistical analysis was performed using one-way ANOVA with Tukey's multiple comparisons test (B and D). *$P < 0.05$, **$P < 0.01$, ***$P < 0.001$.

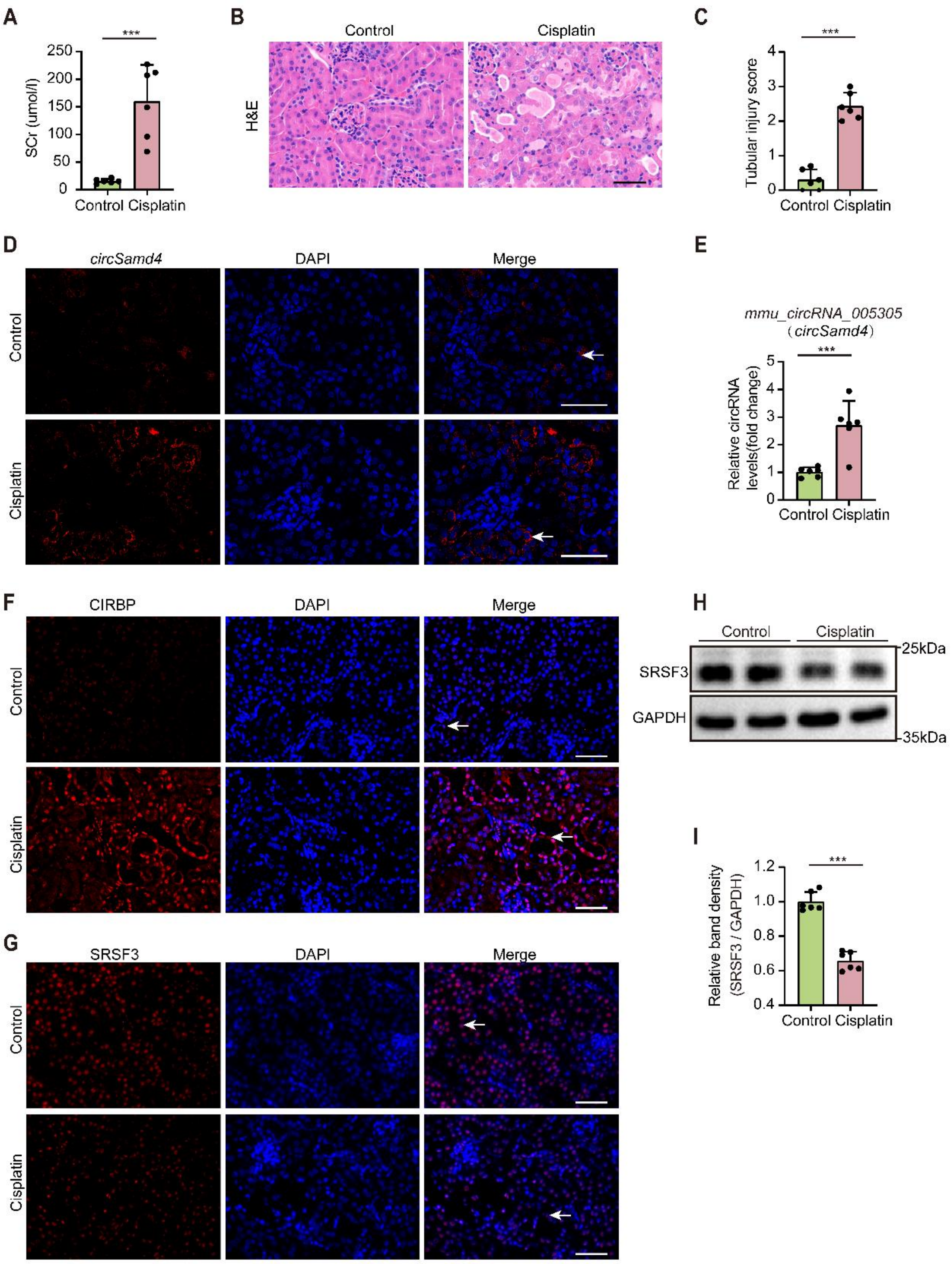
A
***
250
200
150
100
50
0
SCr (umol/l)
Control Cisplatin
B
Control
Cisplatin
H&E
C
***
Tubular injury score
Control Cisplatin
D
circSamd4
DAPI
Merge
Control
Cisplatin
E
mmu_circRNA_005305
(circSamd4)
***
Relative circRNA levels(fold change)
Control Cisplatin
F
CIRBP
DAPI
Merge
Control
Cisplatin
H
Control
Cisplatin
25kDa
SRSF3
GAPDH
35kDa
G
SRSF3
DAPI
Merge
Control
Cisplatin
I
***
Relative band density (SRSF3 / GAPDH)
1.2
1.0
0.8
0.6
0.4
Control Cisplatin

**Figure S9. Altered expression of SRSF3, *circSamd4* and CIRBP in RTECs in cisplatin-induced AKI.**

Male C57BL/6 mice were injected with one dose of cisplatin (30 mg/kg). Control mice were injected with normal saline. Serum and kidney tissues were collected at 48 hours after cisplatin injection. (A) Serum creatinine (SCr) levels (n = 6). (B and C) Representative images of hematoxylin and eosin (H&E) staining and pathological score in kidney tissues (n = 6). Scale bar: 50 μm. (D) Representative RNA FISH staining images of *circSamd4* in kidney tissues. The white arrows indicated *circSamd4*-positive signals. Scale bar: 50 μm. (E) *CircSamd4* (*mmu_circ_005305*) expression in kidney tissues, measured by qRT-PCR (n = 6). (F) Representative immunofluorescence images of CIRBP in kidney sections from mice. Scale bar: 50μm. The arrows point to nuclear staining of CIRBP in control or nuclear and cytoplasmic staining in RTECs in cisplatin-induced AKI. (G) Representative immunofluorescence images of SRSF3 in kidney sections from mice. Scale bar: 50μm. Arrows point to representative tubule cells with nuclear SRSF3. (H-I) Immunoblot and densitometric analyses of SRSF3 expression in kidney cortex (n = 6). All quantitative data are presented as mean ± SD (A, C, E and I). Statistical analysis was performed using unpaired two-tailed Student's t-test (A, C, E and I). ***$P$ < 0.001.

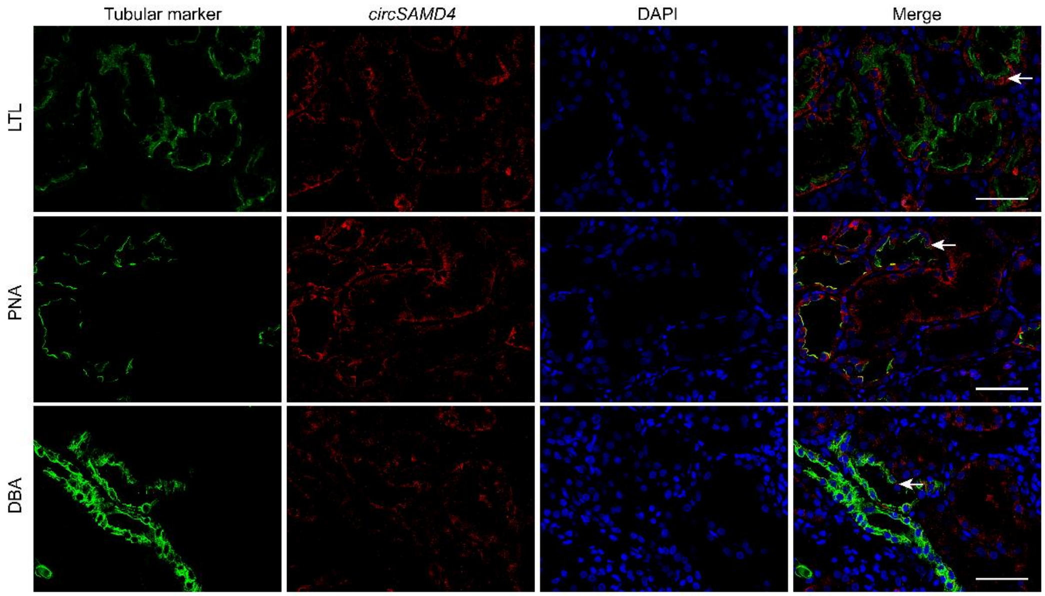


**Figure S10**. Co-localization staining of *circSAMD4* and various segment-specific tubular markers in renal biopsy specimens from patients pathologically diagnosed with ATI. *CircSAMD4* (red) and various segment-specific tubular markers (green), including lotus tetragonolobus lectin (LTL), peanut agglutinin (PNA), and dolichos biflorus agglutinin (DBA), were detected by RNA FISH-immunofluorescence. Scale bar, 50 μm.

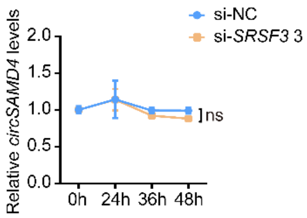


**Figure S11**. qRT-PCR analysis for the expression of *circSAMD4* after treatment with Actinomycin D (10 μg/mL) at the indicated time points in HK-2 cells (n = 4). All quantitative data are presented as mean ± SD. Statistical analysis was performed using two-way ANOVA with Tukey's multiple comparisons test. ns, not significant.
